# Supplementary material for: Inappropriate Evaluation of Effect Modifications Based on Categorical Outcomes: A Systematic Review of Randomized Controlled Trials
Source: Int J Environ Res Public Health. 2022 Nov 18;19(22):15262. doi: 10.3390/ijerph192215262 (PMC9690675; doi:10.3390/ijerph192215262)
Supplement: Supplementary file 1 [file ijerph-19-15262-s001.zip › ijerph-1961809-supplementary.pdf]

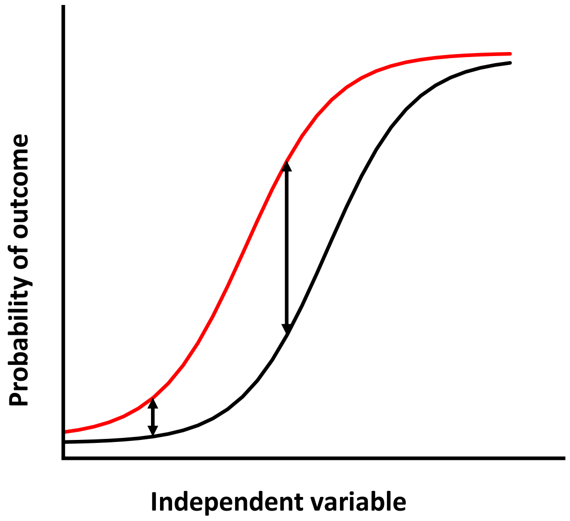

Figure S1. Inherent interactive nature of logistic regression

Table S1. Simulation of evaluating an absolute and relative difference of an interaction effect

	X +	X -
Treatment	16%	8%
Non-treatment	8%	4%

The absolute differences of treatment success rates between treatment and non-treatment are 16-8 = 8% in the X+ group, and 8-4 = 4% in the X- group. It suggests there is an effect modification. On the other hand, the relative differences are 16/8 = 2 in the X+ group, and 8/4 = 2 in the X- group. It suggests there is no effect modification.

Table S2. Search terms used in PubMed (conducted on May 19, 2022)

	Search term
# 1	Randomized controlled trial[PT]
# 2	"N Engl J Med"[Journal]
# 3	"Lancet"[Journal]
# 4	"JAMA"[Journal]
# 5	"BMJ"[Journal]
# 6	"Ann Intern Med"[Journal]
# 7	"JAMA Intern Med"[Journal]
# 8	"Lancet Oncol"[Journal]
# 9	"J Clin Oncol"[Journal]

# 10	"Lancet Neurol"[Journal]
# 11	"Lancet Infect Dis"[Journal]
# 12	#1 AND (#2 OR #3 OR #4 OR #5 OR #6 OR #7 OR #8 OR #8 OR #9 OR #10 OR #11)
# 13	#12 AND (2021:2021[pdat])

Table S3. Summary of evaluation of effect modifications in the included articles

Titles	Citations	Outcome scale	Interpretation of interaction terms
A Neutralizing Monoclonal Antibody for Hospitalized Patients with Covid-19.	The New England journal of medicine 2021;384:10:905-914	Only relative scale	Inappropriate
A Randomized Trial of Intravenous Alteplase before Endovascular Treatment for Stroke.	The New England journal of medicine 2021;385:20:1833-1844	Only relative scale	Inappropriate
Behavioral interventions to improve population health outreach for hepatitis C screening: randomized clinical trial.	BMJ 2021;373:n1022	Only relative scale	Inappropriate
Brief Advice, Nicotine Replacement Therapy Sampling, and Active Referral for Expectant Fathers Who Smoke Cigarettes: A Randomized Clinical Trial.	JAMA internal medicine 2021;181:8:1081-1089	Only relative scale	Inappropriate
Effect of a Digital Intervention on Depressive Symptoms in Patients With Comorbid Hypertension or Diabetes in Brazil and Peru: Two Randomized Clinical Trials.	JAMA 2021;325:18:1852-1862	Only relative scale	Inappropriate
Effect of Continuous Infusion of Hypertonic Saline vs Standard Care on 6-Month Neurological Outcomes in Patients With	JAMA 2021;325:20:2056-	Only relative	Inappropriate

Traumatic Brain Injury: The COBI Randomized Clinical Trial.	2066	scale	
Effect of dexamethasone on complications or all cause mortality after major non-cardiac surgery: multicentre, double blind, randomised controlled trial.	BMJ 2021;373:n1162	Only relative scale	Not applicable
Effect of Lower Tidal Volume Ventilation Facilitated by Extracorporeal Carbon Dioxide Removal vs Standard Care Ventilation on 90-Day Mortality in Patients With Acute Hypoxemic Respiratory Failure: The REST Randomized Clinical Trial.	JAMA 2021;326:11:1013-1023	Only relative scale	Inappropriate
Effect of Minimally Invasive Surfactant Therapy vs Sham Treatment on Death or Bronchopulmonary Dysplasia in Preterm Infants With Respiratory Distress Syndrome: The OPTIMIST-A Randomized Clinical Trial.	JAMA 2021;326:24:2478-2487	Only relative scale	Inappropriate
Effect of Moderate vs Mild Therapeutic Hypothermia on Mortality and Neurologic Outcomes in Comatose Survivors of Out-of-Hospital Cardiac Arrest: The CAPITAL CHILL Randomized Clinical Trial.	JAMA 2021;326:15:1494-1503	Only relative scale	Inappropriate
Effect of Oral Azithromycin vs Placebo on COVID-19 Symptoms in Outpatients With SARS-CoV-2 Infection: A Randomized Clinical Trial.	JAMA 2021;326:6:490-498	Absolute scale	Appropriate
Effect of Salt Substitution on Cardiovascular Events and Death.	The New England journal of medicine 2021;385:12:1067-1077	Only relative scale	Inappropriate

Effect of Slower vs Faster Intravenous Fluid Bolus Rates on Mortality in Critically Ill Patients: The BaSICS Randomized Clinical Trial.	JAMA 2021;326:9:830-838	Only relative scale	Inappropriate
Effect of the STAMP (Sharing and Talking About My Preferences) Intervention on Completing Multiple Advance Care Planning Activities in Ambulatory Care : A Cluster Randomized Controlled Trial.	Annals of internal medicine 2021;174:11:1519-1527	Only relative scale	Inappropriate
Effect of Therapeutic Drug Monitoring vs Standard Therapy During Maintenance Infliximab Therapy on Disease Control in Patients With Immune-Mediated Inflammatory Diseases: A Randomized Clinical Trial.	JAMA 2021;326:23:2375-2384	Absolute scale	Not applicable
Effect of Thrombectomy With Combined Contact Aspiration and Stent Retriever vs Stent Retriever Alone on Revascularization in Patients With Acute Ischemic Stroke and Large Vessel Occlusion: The ASTER2 Randomized Clinical Trial.	JAMA 2021;326:12:1158-1169	Only relative scale	Inappropriate
Effect of Tocilizumab vs Standard Care on Clinical Worsening in Patients Hospitalized With COVID-19 Pneumonia: A Randomized Clinical Trial.	JAMA internal medicine 2021;181:1:24-31	Unclear	Unclear
Effect of Use of a Bougie vs Endotracheal Tube With Stylet on Successful Intubation on the First Attempt Among Critically Ill Patients Undergoing Tracheal Intubation: A Randomized Clinical Trial.	JAMA 2021;326:24:2488-2497	Only relative scale	Inappropriate
Effect of Vitamin C, Thiamine, and Hydrocortisone on Ventilator-	JAMA	Only	Inappropriate

and Vasopressor-Free Days in Patients With Sepsis: The VICTAS Randomized Clinical Trial.	2021;325:8:742-750	relative scale	
Effectiveness of a Vaping Cessation Text Message Program Among Young Adult e-Cigarette Users: A Randomized Clinical Trial.	JAMA internal medicine 2021;181:7:923-930	Only relative scale	Inappropriate
Effectiveness of Adding a Mask Recommendation to Other Public Health Measures to Prevent SARS-CoV-2 Infection in Danish Mask Wearers : A Randomized Controlled Trial.	Annals of internal medicine 2021;174:3:335-343	Only relative scale	Inappropriate
Effectiveness of therapeutic heparin versus prophylactic heparin on death, mechanical ventilation, or intensive care unit admission in moderately ill patients with covid-19 admitted to hospital: RAPID randomised clinical trial.	BMJ 2021;375:n2400	Only relative scale	Inappropriate
Effects of Combined Varenicline With Nicotine Patch and of Extended Treatment Duration on Smoking Cessation: A Randomized Clinical Trial.	JAMA 2021;326:15:1485-1493	Only relative scale	Inappropriate
Effects of Mediterranean Diet or Mindfulness-Based Stress Reduction on Prevention of Small-for-Gestational Age Birth Weights in Newborns Born to At-Risk Pregnant Individuals: The IMPACT BCN Randomized Clinical Trial.	JAMA 2021;326:21:2150-2160	Only relative scale	Inappropriate
E-health StandingTall balance exercise for fall prevention in older people: results of a two year randomised controlled trial.	BMJ 2021;373:n740	Only relative scale	Inappropriate
Electronic health record alerts for acute kidney injury: multicenter,	BMJ 2021;372:m4786	Only	Inappropriate

randomized clinical trial.		relative scale	
Financial incentives for smoking cessation in pregnancy: multicentre randomised controlled trial.	BMJ 2021;375:e065217	Only relative scale	Inappropriate
Hypothermia versus Normothermia after Out-of-Hospital Cardiac Arrest.	The New England journal of medicine 2021;384:24:2283-2294	Only relative scale	Inappropriate
Impact of a Genomic Test on Treatment Decision in a Predominantly African American Population With Favorable-Risk Prostate Cancer: A Randomized Trial.	Journal of clinical oncology 2021;39:15:1660-1670	Only relative scale	Inappropriate
Intracytoplasmic sperm injection versus conventional in-vitro fertilisation in couples with infertility in whom the male partner has normal total sperm count and motility: an open-label, randomised controlled trial.	Lancet 2021;397:10284:1554-1563	Only relative scale	Inappropriate
Long-Term Follow-up of the Italian Flexible Sigmoidoscopy Screening Trial.	Annals of internal medicine 2022;175:1:36-45	Only relative scale	Inappropriate
Midodrine for the Prevention of Vasovagal Syncope : A Randomized Clinical Trial.	Annals of internal medicine 2021;174:10:1349-1356	Only relative scale	Inappropriate

Once-daily tenofovir disoproxil fumarate in treatment-naive Taiwanese patients with chronic hepatitis B and minimally raised alanine aminotransferase (TORCH-B): a multicentre, double-blind, placebo-controlled, parallel-group, randomised trial.	The Lancet. Infectious diseases 2021;21:6:823-833	Only relative scale	Inappropriate
Post-discharge after surgery Virtual Care with Remote Automated Monitoring-1 (PVC-RAM-1) technology versus standard care: randomised controlled trial.	BMJ 2021;374:n2209	Only relative scale	Inappropriate
Posterior left pericardiotomy for the prevention of atrial fibrillation after cardiac surgery: an adaptive, single-centre, single-blind, randomised, controlled trial.	Lancet 2021;398:10316:2075-2083	Only relative scale	Inappropriate
Protection by vaccination of children against typhoid fever with a Vi-tetanus toxoid conjugate vaccine in urban Bangladesh: a cluster-randomised trial.	Lancet 2021;398:10301:675-684	Only relative scale	Inappropriate
Randomized Phase II Trial of Nivolumab With Stereotactic Body Radiotherapy Versus Nivolumab Alone in Metastatic Head and Neck Squamous Cell Carcinoma.	Journal of clinical oncology 2021;39:1:30-37	Only relative scale	Inappropriate
Randomized Phase II Trial of Vincristine-Irinotecan With or Without Temozolomide, in Children and Adults With Relapsed or Refractory Rhabdomyosarcoma: A European Paediatric Soft Tissue Sarcoma Study Group and Innovative Therapies for Children With Cancer Trial.	Journal of clinical oncology 2021;39:27:2979-2990	Only relative scale	Inappropriate
Randomized Trial of Two Induction Therapy Regimens for High-Risk Neuroblastoma: HR-NBL1.5 International Society of	Journal of clinical oncology	Only relative	Inappropriate

Pediatric Oncology European Neuroblastoma Group Study.	2021;39:23:2552-2563	scale	
Reducing surgical site infections in low-income and middle-income countries (FALCON): a pragmatic, multicentre, stratified, randomised controlled trial.	Lancet 2021;398:10312:1687-1699	Only relative scale	Inappropriate
Risk of Overcorrection in Rapid Intermittent Bolus vs Slow Continuous Infusion Therapies of Hypertonic Saline for Patients With Symptomatic Hyponatremia: The SALSA Randomized Clinical Trial.	JAMA internal medicine 2021;181:1:81-92	Only relative scale	Inappropriate
Rituximab versus tocilizumab in anti-TNF inadequate responder patients with rheumatoid arthritis (R4RA): 16-week outcomes of a stratified, biopsy-driven, multicentre, open-label, phase 4 randomised controlled trial.	Lancet 2021;397:10271:305-317	Only relative scale	Inappropriate
Rivaroxaban versus no anticoagulation for post-discharge thromboprophylaxis after hospitalisation for COVID-19 (MICHELLE): an open-label, multicentre, randomised, controlled trial.	Lancet 2022;399:10319:50-59	Only relative scale	Inappropriate
Safety and efficacy of intensive blood pressure lowering after successful endovascular therapy in acute ischaemic stroke (BP-TARGET): a multicentre, open-label, randomised controlled trial.	The Lancet. Neurology 2021;20:4:265-274	Unclear	Unclear
Safety and efficacy of the ChAdOx1 nCoV-19 vaccine (AZD1222) against SARS-CoV-2: an interim analysis of four randomised controlled trials in Brazil, South Africa, and the UK.	Lancet 2021;397:10269:99-111	Only relative scale	Inappropriate
Second asymptomatic carotid surgery trial (ACST-2): a	Lancet	Only	Not

randomised comparison of carotid artery stenting versus carotid endarterectomy.	2021;398:10305:1065-1073	relative scale	applicable
Secondary Antibiotic Prophylaxis for Latent Rheumatic Heart Disease.	The New England journal of medicine 2022;386:3:230-240	Absolute scale	Appropriate
Spinal Anesthesia or General Anesthesia for Hip Surgery in Older Adults.	The New England journal of medicine 2021;385:22:2025-2035	Visual inspection of descriptive numerical data	Not applicable
The clinical and microbiological efficacy of temocillin versus cefotaxime in adults with febrile urinary tract infection, and its effects on the intestinal microbiota: a randomised multicentre clinical trial in Sweden.	The Lancet. Infectious diseases 2022;22:3:390-400	Unclear	Unclear
The effect of BCG revaccination on all-cause mortality beyond infancy: 30-year follow-up of a population-based, double-blind, randomised placebo-controlled trial in Malawi.	The Lancet. Infectious diseases 2021;21:11:1590-1597	Only relative scale	Inappropriate
Tocilizumab in patients admitted to hospital with COVID-19 (RECOVERY): a randomised, controlled, open-label, platform trial.	Lancet 2021;397:10285:1637-1645	Only relative scale	Not applicable
Tympanostomy Tubes or Medical Management for Recurrent Acute Otitis Media.	The New England journal of medicine	Only relative	Inappropriate

	2021;384:19:1789-1799	scale	

Table S4. Lists of the excluded articles after the title and abstract screening (N = 526)

Title	Journals
Wrong publication type (N = 13)	
Association of Cycling With All-Cause and Cardiovascular Disease Mortality Among Persons With Diabetes.	JAMA internal medicine 2021;181:12:1678
Hospital Responses to Incentives in Episode-Based Payment for Joint Surgery: A Controlled Population-Based Study.	JAMA internal medicine 2021;181:7:932-940
Home based rehabilitation after a knee replacement is as effective as physiotherapy.	BMJ 2021;375:n2593
Hypofractionated radiotherapy in locally advanced bladder cancer: an individual patient data meta-analysis of the BC2001 and BCON trials.	The Lancet. Oncology 2021;22:2:246-255
Ipilimumab versus ipilimumab plus anti-PD-1 for metastatic melanoma.	The Lancet. Oncology 2021;22:8:e342
Neoadjuvant Docetaxel, Oxaliplatin, and S-1 in Resectable Advanced Gastric Cancer.	Journal of clinical oncology 2021;39:34:3883-3884
Oral steroids for episodic cluster headache.	The Lancet. Neurology 2021;20:1:19-20
Pembrolizumab monotherapy for high-risk, non-muscle-invasive bladder cancer.	The Lancet. Oncology 2021;22:9:e379
Simple Intervention Motivates Expectant Fathers to Quit Smoking.	JAMA 2021;326:5:378
Sintilimab plus IBI305 for hepatocellular carcinoma.	The Lancet. Oncology 2021;22:9:e386

Stereotactic body radiotherapy for painful spinal metastases.	The Lancet. Oncology 2021;22:9:e384
The Effect of Preconception-Initiated Low-Dose Aspirin on Human Chorionic Gonadotropin-Detected Pregnancy, Pregnancy Loss, and Live Birth : Per Protocol Analysis of a Randomized Trial.	Annals of internal medicine 2021;174:5:595-601
Wrong study design (N = 513)	
(177)Lu-Dotatate plus long-acting octreotide versus high<U+2011>dose long-acting octreotide in patients with midgut neuroendocrine tumours (NETTER-1): final overall survival and long-term safety results from an open-label, randomised, controlled, phase 3 trial.	The Lancet. Oncology 2021;22:12:1752-1763
(18)F-fluciclovine-PET/CT imaging versus conventional imaging alone to guide postprostatectomy salvage radiotherapy for prostate cancer (EMPIRE-1): a single centre, open-label, phase 2/3 randomised controlled trial.	Lancet 2021;397:10288:1895-1904
[(177)Lu]Lu-PSMA-617 versus cabazitaxel in patients with metastatic castration-resistant prostate cancer (TheraP): a randomised, open-label, phase 2 trial.	Lancet 2021;397:10276:797-804
21-Gene Assay to Inform Chemotherapy Benefit in Node-Positive Breast Cancer.	The New England journal of medicine 2021;385:25:2336-2347
A Cluster-Randomized Trial of Hydroxychloroquine for Prevention of Covid-19.	The New England journal of medicine 2021;384:5:417-427
A comparison of sunitinib with cabozantinib, crizotinib, and savolitinib for treatment of advanced papillary renal cell carcinoma: a randomised, open-label, phase 2 trial.	Lancet 2021;397:10275:695-703
A Double-Blind, Randomized, Placebo-Controlled Phase 1 Study of Ad26.ZIKV.001, an Ad26-Vectored Anti-Zika Virus Vaccine.	Annals of internal medicine 2021;174:5:585-594

A Primary Care-Based Cognitive Behavioral Therapy Intervention for Long-Term Opioid Users With Chronic Pain : A Randomized Pragmatic Trial.	Annals of internal medicine 2022;175:1:46-55
A Randomized Trial of a Transglutaminase 2 Inhibitor for Celiac Disease.	The New England journal of medicine 2021;385:1:35-45
A Randomized Trial of Albumin Infusions in Hospitalized Patients with Cirrhosis.	The New England journal of medicine 2021;384:9:808-817
A Randomized Trial of Convalescent Plasma in Covid-19 Severe Pneumonia.	The New England journal of medicine 2021;384:7:619-629
A Trial of Hyperimmune Globulin to Prevent Congenital Cytomegalovirus Infection.	The New England journal of medicine 2021;385:5:436-444
Abrocitinib versus Placebo or Dupilumab for Atopic Dermatitis.	The New England journal of medicine 2021;384:12:1101-1112
Acalabrutinib Versus Ibrutinib in Previously Treated Chronic Lymphocytic Leukemia: Results of the First Randomized Phase III Trial.	Journal of clinical oncology 2021;39:31:3441-3452
Adavosertib plus gemcitabine for platinum-resistant or platinum-refractory recurrent ovarian cancer: a double-blind, randomised, placebo-controlled, phase 2 trial.	Lancet 2021;397:10271:281-292
Addition of Lenalidomide to R-CHOP Improves Outcomes in Newly Diagnosed Diffuse Large B-Cell Lymphoma in a Randomized Phase II US Intergroup Study ECOG-ACRIN E1412.	Journal of clinical oncology 2021;39:12:1329-1338
Adjustable intragastric balloon for treatment of obesity: a multicentre, open-label, randomised clinical	Lancet

trial.	2021;398:10315:1965-1973
Adjuvant atezolizumab versus observation in muscle-invasive urothelial carcinoma (IMvigor010): a multicentre, open-label, randomised, phase 3 trial.	The Lancet. Oncology 2021;22:4:525-537
Adjuvant Nivolumab in Resected Esophageal or Gastroesophageal Junction Cancer.	The New England journal of medicine 2021;384:13:1191-1203
Adjuvant Nivolumab versus Placebo in Muscle-Invasive Urothelial Carcinoma.	The New England journal of medicine 2021;384:22:2102-2114
Adjuvant Olaparib for Patients with BRCA1- or BRCA2-Mutated Breast Cancer.	The New England journal of medicine 2021;384:25:2394-2405
Adjuvant Palbociclib for Early Breast Cancer: The PALLAS Trial Results (ABCSG-42/AFT-05/BIG-14-03).	Journal of clinical oncology 2022;40:3:282-293
Adjuvant Pembrolizumab after Nephrectomy in Renal-Cell Carcinoma.	The New England journal of medicine 2021;385:8:683-694
Adjuvant pembrolizumab versus placebo in resected stage III melanoma (EORTC 1325-MG/KEYNOTE-054): distant metastasis-free survival results from a double-blind, randomised, controlled, phase 3 trial.	The Lancet. Oncology 2021;22:5:643-654
Adjuvant pembrolizumab versus placebo in resected stage III melanoma (EORTC 1325-MG/KEYNOTE-054): health-related quality-of-life results from a double-blind, randomised, controlled, phase 3 trial.	The Lancet. Oncology 2021;22:5:655-664

Adjuvant Pertuzumab and Trastuzumab in Early HER2-Positive Breast Cancer in the APHINITY Trial: 6 Years' Follow-Up.	Journal of clinical oncology 2021;39:13:1448-1457
Adjuvant S-1 plus endocrine therapy for oestrogen receptor-positive, HER2-negative, primary breast cancer: a multicentre, open-label, randomised, controlled, phase 3 trial.	The Lancet. Oncology 2021;22:1:74-84
Adjuvant Trastuzumab Emtansine Versus Paclitaxel in Combination With Trastuzumab for Stage I HER2-Positive Breast Cancer (ATEMPT): A Randomized Clinical Trial.	Journal of clinical oncology 2021;39:21:2375-2385
Angiographic quantitative flow ratio-guided coronary intervention (FAVOR III China): a multicentre, randomised, sham-controlled trial.	Lancet 2021;398:10317:2149-2159
Angiography after Out-of-Hospital Cardiac Arrest without ST-Segment Elevation.	The New England journal of medicine 2021;385:27:2544-2553
Angiotensin Receptor-Neprilysin Inhibition in Acute Myocardial Infarction.	The New England journal of medicine 2021;385:20:1845-1855
Annual Tuberculosis Preventive Therapy for Persons With HIV Infection : A Randomized Trial.	Annals of internal medicine 2021;174:10:1367-1376
Antibiotics for lower respiratory tract infection in children presenting in primary care in England (ARTIC PC): a double-blind, randomised, placebo-controlled trial.	Lancet 2021;398:10309:1417-1426
Antihypertensive treatment and risk of cancer: an individual participant data meta-analysis.	The Lancet. Oncology 2021;22:4:558-570
Antiseptic mouthwash for gonorrhoea prevention (OMEGA): a randomised, double-blind, parallel-group, multicentre trial.	The Lancet. Infectious diseases 2021;21:5:647-656
Apalutamide in Patients With Metastatic Castration-Sensitive Prostate Cancer: Final Survival	Journal of clinical oncology

Analysis of the Randomized, Double-Blind, Phase III TITAN Study.	2021;39:20:2294-2303
Apalutamide plus abiraterone acetate and prednisone versus placebo plus abiraterone and prednisone in metastatic, castration-resistant prostate cancer (ACIS): a randomised, placebo-controlled, double-blind, multinational, phase 3 study.	The Lancet. Oncology 2021;22:11:1541-1559
Apixaban versus no anticoagulation after anticoagulation-associated intracerebral haemorrhage in patients with atrial fibrillation in the Netherlands (APACHE-AF): a randomised, open-label, phase 2 trial.	The Lancet. Neurology 2021;20:11:907-916
Arsenic Combined With All-Trans Retinoic Acid for Pediatric Acute Promyelocytic Leukemia: Report From the CCLG-APL2016 Protocol Study.	Journal of clinical oncology 2021;39:28:3161-3170
Arterial Chemotherapy of Oxaliplatin Plus Fluorouracil Versus Sorafenib in Advanced Hepatocellular Carcinoma: A Biomolecular Exploratory, Randomized, Phase III Trial (FOHAIC-1).	Journal of clinical oncology 2022;40:5:468-480
ARTSCAN III: A Randomized Phase III Study Comparing Chemoradiotherapy With Cisplatin Versus Cetuximab in Patients With Locoregionally Advanced Head and Neck Squamous Cell Cancer.	Journal of clinical oncology 2021;39:1:38-47
Aspirin in patients admitted to hospital with COVID-19 (RECOVERY): a randomised, controlled, open-label, platform trial.	Lancet 2022;399:10320:143-151
Aspirin versus anticoagulation in cervical artery dissection (TREAT-CAD): an open-label, randomised, non-inferiority trial.	The Lancet. Neurology 2021;20:5:341-350
Aspirin versus clopidogrel for chronic maintenance monotherapy after percutaneous coronary intervention (HOST-EXAM): an investigator-initiated, prospective, randomised, open-label, multicentre trial.	Lancet 2021;397:10293:2487-2496
Atezolizumab, Bevacizumab, and Chemotherapy for Newly Diagnosed Stage III or IV Ovarian Cancer: Placebo-Controlled Randomized Phase III Trial (IMagyn050/GOG 3015/ENGOT-OV39).	Journal of clinical oncology 2021;39:17:1842-1855
Augmented Reduced-Intensity Regimen Does Not Improve Postallogeneic Transplant Outcomes in	Journal of clinical oncology

Acute Myeloid Leukemia.	2021;39:7:768-778
Avacopan for the Treatment of ANCA-Associated Vasculitis.	The New England journal of medicine 2021;384:7:599-609
Avapritinib Versus Regorafenib in Locally Advanced Unresectable or Metastatic GI Stromal Tumor: A Randomized, Open-Label Phase III Study.	Journal of clinical oncology 2021;39:28:3128-3139
Avelumab plus standard-of-care chemoradiotherapy versus chemoradiotherapy alone in patients with locally advanced squamous cell carcinoma of the head and neck: a randomised, double-blind, placebo-controlled, multicentre, phase 3 trial.	The Lancet. Oncology 2021;22:4:450-462
Axicabtagene Ciloleucel as Second-Line Therapy for Large B-Cell Lymphoma.	The New England journal of medicine 2022;386:7:640-654
Azithromycin for community treatment of suspected COVID-19 in people at increased risk of an adverse clinical course in the UK (PRINCIPLE): a randomised, controlled, open-label, adaptive platform trial.	Lancet 2021;397:10279:1063-1074
Azithromycin in patients admitted to hospital with COVID-19 (RECOVERY): a randomised, controlled, open-label, platform trial.	Lancet 2021;397:10274:605-612
Azithromycin or Doxycycline for Asymptomatic Rectal Chlamydia trachomatis.	The New England journal of medicine 2021;384:25:2418-2427
Bah humbug! Association between sending Christmas cards to trial participants and trial retention: randomised study within a trial conducted simultaneously across eight host trials.	BMJ 2021;375:e067742
Bamlanivimab plus Etesevimab in Mild or Moderate Covid-19.	The New England journal of

	medicine 2021;385:15:1382-1392
Baricitinib plus Remdesivir for Hospitalized Adults with Covid-19.	The New England journal of medicine 2021;384:9:795-807
BCG-induced non-specific effects on heterologous infectious disease in Ugandan neonates: an investigator-blind randomised controlled trial.	The Lancet. Infectious diseases 2021;21:7:993-1003
Bimekizumab efficacy and safety in moderate to severe plaque psoriasis (BE READY): a multicentre, double-blind, placebo-controlled, randomised withdrawal phase 3 trial.	Lancet 2021;397:10273:475-486
Bimekizumab versus Secukinumab in Plaque Psoriasis.	The New England journal of medicine 2021;385:2:142-152
Biomarker-guided tuberculosis preventive therapy (CORTIS): a randomised controlled trial.	The Lancet. Infectious diseases 2021;21:3:354-365
BRE12-158: A Postneoadjuvant, Randomized Phase II Trial of Personalized Therapy Versus Treatment of Physician's Choice for Patients With Residual Triple-Negative Breast Cancer.	Journal of clinical oncology 2022;40:4:345-355
Bupropion and Naltrexone in Methamphetamine Use Disorder.	The New England journal of medicine 2021;384:2:140-153
Cabozantinib for radioiodine-refractory differentiated thyroid cancer (COSMIC-311): a randomised, double-blind, placebo-controlled, phase 3 trial.	The Lancet. Oncology 2021;22:8:1126-1138
Cabozantinib in Combination With Atezolizumab for Advanced Renal Cell Carcinoma: Results From the COSMIC-021 Study.	Journal of clinical oncology 2021;39:33:3725-3736

Camrelizumab versus placebo in combination with gemcitabine and cisplatin as first-line treatment for recurrent or metastatic nasopharyngeal carcinoma (CAPTAIN-1st): a multicentre, randomised, double-blind, phase 3 trial.	The Lancet. Oncology 2021;22:8:1162-1174
CanDirect: Effectiveness of a Telephone-Supported Depression Self-Care Intervention for Cancer Survivors.	Journal of clinical oncology 2021;39:10:1150-1161
Capecitabine Versus Active Monitoring in Stable or Responding Metastatic Colorectal Cancer After 16 Weeks of First-Line Therapy: Results of the Randomized FOCUS4-N Trial.	Journal of clinical oncology 2021;39:33:3693-3704
Carboplatin-based doublet plus bevacizumab beyond progression versus carboplatin-based doublet alone in patients with platinum-sensitive ovarian cancer: a randomised, phase 3 trial.	The Lancet. Oncology 2021;22:2:267-276
Cardiac Myosin Activation with Omecamtiv Mecarbil in Systolic Heart Failure.	The New England journal of medicine 2021;384:2:105-116
Cardiovascular Events with Finerenone in Kidney Disease and Type 2 Diabetes.	The New England journal of medicine 2021;385:24:2252-2263
Carfilzomib, dexamethasone, and daratumumab versus carfilzomib and dexamethasone for patients with relapsed or refractory multiple myeloma (CANDOR): updated outcomes from a randomised, multicentre, open-label, phase 3 study.	The Lancet. Oncology 2022;23:1:65-76
Cefiderocol versus high-dose, extended-infusion meropenem for the treatment of Gram-negative nosocomial pneumonia (APEKS-NP): a randomised, double-blind, phase 3, non-inferiority trial.	The Lancet. Infectious diseases 2021;21:2:213-225
Cemiplimab monotherapy for first-line treatment of advanced non-small-cell lung cancer with PD-L1 of at least 50%: a multicentre, open-label, global, phase 3, randomised, controlled trial.	Lancet 2021;397:10274:592-604
Central venous access devices for the delivery of systemic anticancer therapy (CAVA): a randomised	Lancet 2021;398:10298:403-

controlled trial.	415
Cetuximab, docetaxel, and cisplatin versus platinum, fluorouracil, and cetuximab as first-line treatment in patients with recurrent or metastatic head and neck squamous-cell carcinoma (GORTEC 2014-01 TPExtreme): a multicentre, open-label, randomised, phase 2 trial.	The Lancet. Oncology 2021;22:4:463-475
Chemotherapy de-escalation using an (18)F-FDG-PET-based pathological response-adapted strategy in patients with HER2-positive early breast cancer (PHERGain): a multicentre, randomised, open-label, non-comparative, phase 2 trial.	The Lancet. Oncology 2021;22:6:858-871
Children's Oncology Group Phase III Trial of Reduced-Dose and Reduced-Volume Radiotherapy With Chemotherapy for Newly Diagnosed Average-Risk Medulloblastoma.	Journal of clinical oncology 2021;39:24:2685-2697
Chlorhexidine plus alcohol versus povidone iodine plus alcohol, combined or not with innovative devices, for prevention of short-term peripheral venous catheter infection and failure (CLEAN 3 study): an investigator-initiated, open-label, single centre, randomised-controlled, two-by-two factorial trial.	The Lancet. Infectious diseases 2021;21:7:1038-1048
Chlorthalidone for Hypertension in Advanced Chronic Kidney Disease.	The New England journal of medicine 2021;385:27:2507-2519
Circulating tumour DNA in patients with advanced melanoma treated with dabrafenib or dabrafenib plus trametinib: a clinical validation study.	The Lancet. Oncology 2021;22:3:370-380
Clinical outcomes in systematic screening for atrial fibrillation (STROKESTOP): a multicentre, parallel group, unmasked, randomised controlled trial.	Lancet 2021;398:10310:1498-1506
Clinical Responses of Oncolytic Coxsackievirus A21 (V937) in Patients With Unresectable Melanoma.	Journal of clinical oncology 2021;39:34:3829-3838
Closed incision negative pressure wound therapy versus standard dressings in obese women	BMJ 2021;373:n893

undergoing caesarean section: multicentre parallel group randomised controlled trial.	
Comparing real-time and intermittently scanned continuous glucose monitoring in adults with type 1 diabetes (ALERTT1): a 6-month, prospective, multicentre, randomised controlled trial.	Lancet 2021;397:10291:2275-2283
Comparison of Knowledge and Information-Seeking Behavior After General COVID-19 Public Health Messages and Messages Tailored for Black and Latinx Communities : A Randomized Controlled Trial.	Annals of internal medicine 2021;174:4:484-492
Comparison of Radiation With or Without Concurrent Trastuzumab for HER2-Positive Ductal Carcinoma In Situ Resected by Lumpectomy: A Phase III Clinical Trial.	Journal of clinical oncology 2021;39:21:2367-2374
Comparison of the safety and immunogenicity of a novel Matrix-M-adjuvanted nanoparticle influenza vaccine with a quadrivalent seasonal influenza vaccine in older adults: a phase 3 randomised controlled trial.	The Lancet. Infectious diseases 2022;22:1:73-84
Comparison of two delayed strategies for renal replacement therapy initiation for severe acute kidney injury (AKIKI 2): a multicentre, open-label, randomised, controlled trial.	Lancet 2021;397:10281:1293-1300
Concomitant Tricuspid Repair in Patients with Degenerative Mitral Regurgitation.	The New England journal of medicine 2022;386:4:327-339
Consolidation and Maintenance in Newly Diagnosed Multiple Myeloma.	Journal of clinical oncology 2021;39:32:3613-3622
Continued versus discontinued oxytocin stimulation in the active phase of labour (CONDISOX): double blind randomised controlled trial.	BMJ 2021;373:n716
Convalescent plasma in patients admitted to hospital with COVID-19 (RECOVERY): a randomised controlled, open-label, platform trial.	Lancet 2021;397:10289:2049-2059
Copanlisib plus rituximab versus placebo plus rituximab in patients with relapsed indolent non-	The Lancet. Oncology

Hodgkin lymphoma (CHRONOS-3): a double-blind, randomised, placebo-controlled, phase 3 trial.	2021;22:5:678-689
Cryoablation or Drug Therapy for Initial Treatment of Atrial Fibrillation.	The New England journal of medicine 2021;384:4:305-315
Cryoballoon Ablation as Initial Therapy for Atrial Fibrillation.	The New England journal of medicine 2021;384:4:316-324
Cytoreductive surgery plus hyperthermic intraperitoneal chemotherapy versus cytoreductive surgery alone for colorectal peritoneal metastases (PRODIGE 7): a multicentre, randomised, open-label, phase 3 trial.	The Lancet. Oncology 2021;22:2:256-266
Daily testing for contacts of individuals with SARS-CoV-2 infection and attendance and SARS-CoV-2 transmission in English secondary schools and colleges: an open-label, cluster-randomised trial.	Lancet 2021;398:10307:1217-1229
Daprodustat for the Treatment of Anemia in Patients Not Undergoing Dialysis.	The New England journal of medicine 2021;385:25:2313-2324
Daratumumab plus pomalidomide and dexamethasone versus pomalidomide and dexamethasone alone in previously treated multiple myeloma (APOLLO): an open-label, randomised, phase 3 trial.	The Lancet. Oncology 2021;22:6:801-812
Daratumumab, lenalidomide, and dexamethasone versus lenalidomide and dexamethasone alone in newly diagnosed multiple myeloma (MAIA): overall survival results from a randomised, open-label, phase 3 trial.	The Lancet. Oncology 2021;22:11:1582-1596
Decompression with or without Fusion in Degenerative Lumbar Spondylolisthesis.	The New England journal of medicine 2021;385:6:526-538

Defining the Optimal Total Number of Chemotherapy Courses in Younger Patients With Acute Myeloid Leukemia: A Comparison of Three Versus Four Courses.	Journal of clinical oncology 2021;39:8:890-901
Dexamethasone and Surgical-Site Infection.	The New England journal of medicine 2021;384:18:1731-1741
Dexmedetomidine or Propofol for Sedation in Mechanically Ventilated Adults with Sepsis.	The New England journal of medicine 2021;384:15:1424-1436
Dietary alteration of n-3 and n-6 fatty acids for headache reduction in adults with migraine: randomized controlled trial.	BMJ 2021;374:n1448
Dolutegravir or Darunavir in Combination with Zidovudine or Tenofovir to Treat HIV.	The New England journal of medicine 2021;385:4:330-341
Donafenib Versus Sorafenib in First-Line Treatment of Unresectable or Metastatic Hepatocellular Carcinoma: A Randomized, Open-Label, Parallel-Controlled Phase II-III Trial.	Journal of clinical oncology 2021;39:27:3002-3011
Dual Antiplatelet Therapy after PCI in Patients at High Bleeding Risk.	The New England journal of medicine 2021;385:18:1643-1655
Dual PD-1 and CTLA-4 Checkpoint Blockade Using Balstilimab and Zalifrelimab Combination as Second-Line Treatment for Advanced Cervical Cancer: An Open-Label Phase II Study.	Journal of clinical oncology 2022;40:7:762-771
Dupilumab in Children with Uncontrolled Moderate-to-Severe Asthma.	The New England journal of medicine 2021;385:24:2230-2240

Duration of Adjuvant Aromatase-Inhibitor Therapy in Postmenopausal Breast Cancer.	The New England journal of medicine 2021;385:5:395-405
Duration of Adjuvant Doublet Chemotherapy (3 or 6 months) in Patients With High-Risk Stage II Colorectal Cancer.	Journal of clinical oncology 2021;39:6:631-641
Durvalumab, with or without tremelimumab, plus platinum-etoposide versus platinum-etoposide alone in first-line treatment of extensive-stage small-cell lung cancer (CASPIAN): updated results from a randomised, controlled, open-label, phase 3 trial.	The Lancet. Oncology 2021;22:1:51-65
E2112: Randomized Phase III Trial of Endocrine Therapy Plus Entinostat or Placebo in Hormone Receptor-Positive Advanced Breast Cancer. A Trial of the ECOG-ACRIN Cancer Research Group.	Journal of clinical oncology 2021;39:28:3171-3181
Early computed tomography coronary angiography in patients with suspected acute coronary syndrome: randomised controlled trial.	BMJ 2021;374:n2106
Early Convalescent Plasma for High-Risk Outpatients with Covid-19.	The New England journal of medicine 2021;385:21:1951-1960
Early Interdisciplinary Supportive Care in Patients With Previously Untreated Metastatic Esophagogastric Cancer: A Phase III Randomized Controlled Trial.	Journal of clinical oncology 2021;39:7:748-756
Early Remdesivir to Prevent Progression to Severe Covid-19 in Outpatients.	The New England journal of medicine 2022;386:4:305-315
Early surgical reconstruction versus rehabilitation with elective delayed reconstruction for patients with anterior cruciate ligament rupture: COMPARE randomised controlled trial.	BMJ 2021;372:n375
Early Treatment for Covid-19 with SARS-CoV-2 Neutralizing Antibody Sotrovimab.	The New England journal of

	medicine 2021;385:21:1941-1950
Edoxaban versus Vitamin K Antagonist for Atrial Fibrillation after TAVR.	The New England journal of medicine 2021;385:23:2150-2160
Effect of 12 mg vs 6 mg of Dexamethasone on the Number of Days Alive Without Life Support in Adults With COVID-19 and Severe Hypoxemia: The COVID STEROID 2 Randomized Trial.	JAMA 2021;326:18:1807-1817
Effect of 2 Inactivated SARS-CoV-2 Vaccines on Symptomatic COVID-19 Infection in Adults: A Randomized Clinical Trial.	JAMA 2021;326:1:35-45
Effect of a Diagnostic Strategy Using an Elevated and Age-Adjusted D-Dimer Threshold on Thromboembolic Events in Emergency Department Patients With Suspected Pulmonary Embolism: A Randomized Clinical Trial.	JAMA 2021;326:21:2141-2149
Effect of a Hospital and Postdischarge Quality Improvement Intervention on Clinical Outcomes and Quality of Care for Patients With Heart Failure With Reduced Ejection Fraction: The CONNECT-HF Randomized Clinical Trial.	JAMA 2021;326:4:314-323
Effect of a Multifaceted Clinical Pharmacist Intervention on Medication Safety After Hospitalization in Persons Prescribed High-risk Medications: A Randomized Clinical Trial.	JAMA internal medicine 2021;181:5:610-618
Effect of a Pulmonary Embolism Diagnostic Strategy on Clinical Outcomes in Patients Hospitalized for COPD Exacerbation: A Randomized Clinical Trial.	JAMA 2021;326:13:1277-1285
Effect of a Restrictive vs Liberal Blood Transfusion Strategy on Major Cardiovascular Events Among Patients With Acute Myocardial Infarction and Anemia: The REALITY Randomized Clinical Trial.	JAMA 2021;325:6:552-560
Effect of a Sedation and Ventilator Liberation Protocol vs Usual Care on Duration of Invasive Mechanical Ventilation in Pediatric Intensive Care Units: A Randomized Clinical Trial.	JAMA 2021;326:5:401-410

Effect of a Single High Dose of Vitamin D3 on Hospital Length of Stay in Patients With Moderate to Severe COVID-19: A Randomized Clinical Trial.	JAMA 2021;325:11:1053-1060
Effect of Amoxicillin Dose and Treatment Duration on the Need for Antibiotic Re-treatment in Children With Community-Acquired Pneumonia: The CAP-IT Randomized Clinical Trial.	JAMA 2021;326:17:1713-1724
Effect of an Oncology Nurse-Led Primary Palliative Care Intervention on Patients With Advanced Cancer: The CONNECT Cluster Randomized Clinical Trial.	JAMA internal medicine 2021;181:11:1451-1460
Effect of Antimicrobial Therapy on Respiratory Hospitalization or Death in Adults With Idiopathic Pulmonary Fibrosis: The CleanUP-IPF Randomized Clinical Trial.	JAMA 2021;325:18:1841-1851
Effect of Bamlanivimab as Monotherapy or in Combination With Etesevimab on Viral Load in Patients With Mild to Moderate COVID-19: A Randomized Clinical Trial.	JAMA 2021;325:7:632-644
Effect of Bamlanivimab vs Placebo on Incidence of COVID-19 Among Residents and Staff of Skilled Nursing and Assisted Living Facilities: A Randomized Clinical Trial.	JAMA 2021;326:1:46-55
Effect of Bedside Compared With Outside the Room Patient Case Presentation on Patients' Knowledge About Their Medical Care : A Randomized, Controlled, Multicenter Trial.	Annals of internal medicine 2021;174:9:1282-1292
Effect of Behavioral Therapy With In-Clinic or Telephone Group Visits vs In-Clinic Individual Visits on Weight Loss Among Patients With Obesity in Rural Clinical Practice: A Randomized Clinical Trial.	JAMA 2021;325:4:363-372
Effect of Blinatumomab vs Chemotherapy on Event-Free Survival Among Children With High-risk First-Relapse B-Cell Acute Lymphoblastic Leukemia: A Randomized Clinical Trial.	JAMA 2021;325:9:843-854
Effect of C reactive protein point-of-care testing on antibiotic prescribing for lower respiratory tract infections in nursing home residents: cluster randomised controlled trial.	BMJ 2021;374:n2198
Effect of Camrelizumab vs Placebo Added to Chemotherapy on Survival and Progression-Free Survival in Patients With Advanced or Metastatic Esophageal Squamous Cell Carcinoma: The	JAMA 2021;326:10:916-925

ESCORT-1st Randomized Clinical Trial.	
Effect of Canakinumab vs Placebo on Survival Without Invasive Mechanical Ventilation in Patients Hospitalized With Severe COVID-19: A Randomized Clinical Trial.	JAMA 2021;326:3:230-239
Effect of Capecitabine Maintenance Therapy Using Lower Dosage and Higher Frequency vs Observation on Disease-Free Survival Among Patients With Early-Stage Triple-Negative Breast Cancer Who Had Received Standard Treatment: The SYSUCC-001 Randomized Clinical Trial.	JAMA 2021;325:1:50-58
Effect of Celecoxib vs Placebo Added to Standard Adjuvant Therapy on Disease-Free Survival Among Patients With Stage III Colon Cancer: The CALGB/SWOG 80702 (Alliance) Randomized Clinical Trial.	JAMA 2021;325:13:1277-1286
Effect of Continued Weekly Subcutaneous Semaglutide vs Placebo on Weight Loss Maintenance in Adults With Overweight or Obesity: The STEP 4 Randomized Clinical Trial.	JAMA 2021;325:14:1414-1425
Effect of Continuous Glucose Monitoring on Glycemic Control in Patients With Type 2 Diabetes Treated With Basal Insulin: A Randomized Clinical Trial.	JAMA 2021;325:22:2262-2272
Effect of Convalescent Plasma on Organ Support-Free Days in Critically Ill Patients With COVID-19: A Randomized Clinical Trial.	JAMA 2021;326:17:1690-1702
Effect of dietary sources of calcium and protein on hip fractures and falls in older adults in residential care: cluster randomised controlled trial.	BMJ 2021;375:n2364
Effect of Discontinuing vs Continuing Angiotensin-Converting Enzyme Inhibitors and Angiotensin II Receptor Blockers on Days Alive and Out of the Hospital in Patients Admitted With COVID-19: A Randomized Clinical Trial.	JAMA 2021;325:3:254-264
Effect of general practitioner-led versus surgeon-led colon cancer survivorship care, with or without eHealth support, on quality of life (I CARE): an interim analysis of 1-year results of a randomised, controlled trial.	The Lancet. Oncology 2021;22:8:1175-1187

Effect of Half-Dose vs Stable-Dose Conventional Synthetic Disease-Modifying Antirheumatic Drugs on Disease Flares in Patients With Rheumatoid Arthritis in Remission: The ARCTIC REWIND Randomized Clinical Trial.	JAMA 2021;325:17:1755-1764
Effect of Helmet Noninvasive Ventilation vs High-Flow Nasal Oxygen on Days Free of Respiratory Support in Patients With COVID-19 and Moderate to Severe Hypoxemic Respiratory Failure: The HENIVOT Randomized Clinical Trial.	JAMA 2021;325:17:1731-1743
Effect of High-Dose Baclofen on Agitation-Related Events Among Patients With Unhealthy Alcohol Use Receiving Mechanical Ventilation: A Randomized Clinical Trial.	JAMA 2021;325:8:732-741
Effect of High-Dose Trivalent vs Standard-Dose Quadrivalent Influenza Vaccine on Mortality or Cardiopulmonary Hospitalization in Patients With High-risk Cardiovascular Disease: A Randomized Clinical Trial.	JAMA 2021;325:1:39-49
Effect of High-Flow Oxygen Therapy vs Conventional Oxygen Therapy on Invasive Mechanical Ventilation and Clinical Recovery in Patients With Severe COVID-19: A Randomized Clinical Trial.	JAMA 2021;326:21:2161-2171
Effect of Implantable vs Prolonged External Electrocardiographic Monitoring on Atrial Fibrillation Detection in Patients With Ischemic Stroke: The PER DIEM Randomized Clinical Trial.	JAMA 2021;325:21:2160-2168
Effect of infusion set replacement intervals on catheter-related bloodstream infections (RSVP): a randomised, controlled, equivalence (central venous access device)-non-inferiority (peripheral arterial catheter) trial.	Lancet 2021;397:10283:1447-1458
Effect of Intra-articular Platelet-Rich Plasma vs Placebo Injection on Pain and Medial Tibial Cartilage Volume in Patients With Knee Osteoarthritis: The RESTORE Randomized Clinical Trial.	JAMA 2021;326:20:2021-2030
Effect of Intravenous or Intraosseous Calcium vs Saline on Return of Spontaneous Circulation in Adults With Out-of-Hospital Cardiac Arrest: A Randomized Clinical Trial.	JAMA 2021;326:22:2268-2276
Effect of Ivermectin on Time to Resolution of Symptoms Among Adults With Mild COVID-19: A	JAMA 2021;325:14:1426-

Randomized Clinical Trial.	1435
Effect of Long-term Continuous Cardiac Monitoring vs Usual Care on Detection of Atrial Fibrillation in Patients With Stroke Attributed to Large- or Small-Vessel Disease: The STROKE-AF Randomized Clinical Trial.	JAMA 2021;325:21:2169-2177
Effect of Long-term Supplementation With Marine Omega-3 Fatty Acids vs Placebo on Risk of Depression or Clinically Relevant Depressive Symptoms and on Change in Mood Scores: A Randomized Clinical Trial.	JAMA 2021;326:23:2385-2394
Effect of Low-Normal vs High-Normal Oxygenation Targets on Organ Dysfunction in Critically Ill Patients: A Randomized Clinical Trial.	JAMA 2021;326:10:940-948
Effect of Marine Omega-3 Fatty Acid and Vitamin D Supplementation on Incident Atrial Fibrillation: A Randomized Clinical Trial.	JAMA 2021;325:11:1061-1073
Effect of Oral Moxifloxacin vs Intravenous Ertapenem Plus Oral Levofloxacin for Treatment of Uncomplicated Acute Appendicitis: The APPAC II Randomized Clinical Trial.	JAMA 2021;325:4:353-362
Effect of Osteopathic Manipulative Treatment vs Sham Treatment on Activity Limitations in Patients With Nonspecific Subacute and Chronic Low Back Pain: A Randomized Clinical Trial.	JAMA internal medicine 2021;181:5:620-630
Effect of PEP flute self-care versus usual care in early covid-19: non-drug, open label, randomised controlled trial in a Danish community setting.	BMJ 2021;375:e066952
Effect of Platelet-Rich Plasma Injection vs Sham Injection on Tendon Dysfunction in Patients With Chronic Midportion Achilles Tendinopathy: A Randomized Clinical Trial.	JAMA 2021;326:2:137-144
Effect of Platelet-Rich Plasma Injections vs Placebo on Ankle Symptoms and Function in Patients With Ankle Osteoarthritis: A Randomized Clinical Trial.	JAMA 2021;326:16:1595-1605
Effect of Poloxamer 188 vs Placebo on Painful Vaso-Occlusive Episodes in Children and Adults With Sickle Cell Disease: A Randomized Clinical Trial.	JAMA 2021;325:15:1513-1523

Effect of Postreinduction Therapy Consolidation With Blinatumomab vs Chemotherapy on Disease-Free Survival in Children, Adolescents, and Young Adults With First Relapse of B-Cell Acute Lymphoblastic Leukemia: A Randomized Clinical Trial.	JAMA 2021;325:9:833-842
Effect of Probiotics on Incident Ventilator-Associated Pneumonia in Critically Ill Patients: A Randomized Clinical Trial.	JAMA 2021;326:11:1024-1033
Effect of Prophylactic Subcutaneous Scopolamine Butylbromide on Death Rattle in Patients at the End of Life: The SILENCE Randomized Clinical Trial.	JAMA 2021;326:13:1268-1276
Effect of Recombinant Human Granulocyte Colony-Stimulating Factor for Patients With Coronavirus Disease 2019 (COVID-19) and Lymphopenia: A Randomized Clinical Trial.	JAMA internal medicine 2021;181:1:71-78
Effect of Reduced-Dose vs High-Dose Glucocorticoids Added to Rituximab on Remission Induction in ANCA-Associated Vasculitis: A Randomized Clinical Trial.	JAMA 2021;325:21:2178-2187
Effect of Sacubitril/Valsartan vs Standard Medical Therapies on Plasma NT-proBNP Concentration and Submaximal Exercise Capacity in Patients With Heart Failure and Preserved Ejection Fraction: The PARALLAX Randomized Clinical Trial.	JAMA 2021;326:19:1919-1929
Effect of School Integrated Pest Management or Classroom Air Filter Purifiers on Asthma Symptoms in Students With Active Asthma: A Randomized Clinical Trial.	JAMA 2021;326:9:839-850
Effect of screening by clinical breast examination on breast cancer incidence and mortality after 20 years: prospective, cluster randomised controlled trial in Mumbai.	BMJ 2021;372:n256
Effect of Sotagliflozin on Total Hospitalizations in Patients With Type 2 Diabetes and Worsening Heart Failure : A Randomized Trial.	Annals of internal medicine 2021;174:8:1065-1072
Effect of Subcutaneous Semaglutide vs Placebo as an Adjunct to Intensive Behavioral Therapy on Body Weight in Adults With Overweight or Obesity: The STEP 3 Randomized Clinical Trial.	JAMA 2021;325:14:1403-1413
Effect of Telephone Cognitive Behavioral Therapy for Insomnia in Older Adults With Osteoarthritis	JAMA internal medicine

Pain: A Randomized Clinical Trial.	2021;181:4:530-538
Effect of tocilizumab on clinical outcomes at 15 days in patients with severe or critical coronavirus disease 2019: randomised controlled trial.	BMJ 2021;372:n84
Effect of Tocilizumab vs Usual Care in Adults Hospitalized With COVID-19 and Moderate or Severe Pneumonia: A Randomized Clinical Trial.	JAMA internal medicine 2021;181:1:32-40
Effect of Treating Hyperphosphatemia With Lanthanum Carbonate vs Calcium Carbonate on Cardiovascular Events in Patients With Chronic Kidney Disease Undergoing Hemodialysis: The LANDMARK Randomized Clinical Trial.	JAMA 2021;325:19:1946-1954
Effect of Urate-Elevating Inosine on Early Parkinson Disease Progression: The SURE-PD3 Randomized Clinical Trial.	JAMA 2021;326:10:926-939
Effect of Vasopressin and Methylprednisolone vs Placebo on Return of Spontaneous Circulation in Patients With In-Hospital Cardiac Arrest: A Randomized Clinical Trial.	JAMA 2021;326:16:1586-1594
Effect of Ventral vs Dorsal Spinal Surgery on Patient-Reported Physical Functioning in Patients With Cervical Spondylotic Myelopathy: A Randomized Clinical Trial.	JAMA 2021;325:10:942-951
Effectiveness and cost-effectiveness of reactive, targeted indoor residual spraying for malaria control in low-transmission settings: a cluster-randomised, non-inferiority trial in South Africa.	Lancet 2021;397:10276:816-827
Effectiveness of a killed whole-cell oral cholera vaccine in Bangladesh: further follow-up of a cluster-randomised trial.	The Lancet. Infectious diseases 2021;21:10:1407-1414
Effectiveness of a Nurse-Led Multidisciplinary Intervention vs Usual Care on Advance Care Planning for Vulnerable Older Adults in an Accountable Care Organization: A Randomized Clinical Trial.	JAMA internal medicine 2021;181:3:361-369
Effectiveness of a tailored intervention to reduce antibiotics for urinary tract infections in nursing home residents: a cluster, randomised controlled trial.	The Lancet. Infectious diseases 2021;21:11:1549-

	1556
Effectiveness of App-Delivered, Tailored Self-management Support for Adults With Lower Back Pain-Related Disability: A selfBACK Randomized Clinical Trial.	JAMA internal medicine 2021;181:10:1288-1296
Effectiveness of Belimumab After Rituximab in Systemic Lupus Erythematosus : A Randomized Controlled Trial.	Annals of internal medicine 2021;174:12:1647-1657
Effectiveness of Mindfulness Meditation vs Headache Education for Adults With Migraine: A Randomized Clinical Trial.	JAMA internal medicine 2021;181:3:317-328
Effects of a Self-directed Web-Based Strengthening Exercise and Physical Activity Program Supported by Automated Text Messages for People With Knee Osteoarthritis: A Randomized Clinical Trial.	JAMA internal medicine 2021;181:6:776-785
Effects of Intravenous Eptinezumab vs Placebo on Headache Pain and Most Bothersome Symptom When Initiated During a Migraine Attack: A Randomized Clinical Trial.	JAMA 2021;325:23:2348-2356
Efficacy and safety of an inactivated whole-virion SARS-CoV-2 vaccine (CoronaVac): interim results of a double-blind, randomised, placebo-controlled, phase 3 trial in Turkey.	Lancet 2021;398:10296:213-222
Efficacy and safety of cefiderocol or best available therapy for the treatment of serious infections caused by carbapenem-resistant Gram-negative bacteria (CREDIBLE-CR): a randomised, open-label, multicentre, pathogen-focused, descriptive, phase 3 trial.	The Lancet. Infectious diseases 2021;21:2:226-240
Efficacy and safety of co-administered ivermectin and albendazole in school-aged children and adults infected with Trichuris trichiura in Cote d'Ivoire, Laos, and Pemba Island, Tanzania: a double-blind, parallel-group, phase 3, randomised controlled trial.	The Lancet. Infectious diseases 2022;22:1:123-135
Efficacy and safety of dolutegravir with emtricitabine and tenofovir alafenamide fumarate or tenofovir disoproxil fumarate, and efavirenz, emtricitabine, and tenofovir disoproxil fumarate HIV antiretroviral therapy regimens started in pregnancy (IMPAACT 2010/VESTED): a multicentre, open-	Lancet 2021;397:10281:1276-1292

label, randomised, controlled, phase 3 trial.	
Efficacy and Safety of Hydroxychloroquine vs Placebo for Pre-exposure SARS-CoV-2 Prophylaxis Among Health Care Workers: A Randomized Clinical Trial.	JAMA internal medicine 2021;181:2:195-202
Efficacy and safety of maralixibat treatment in patients with Alagille syndrome and cholestatic pruritus (ICONIC): a randomised phase 2 study.	Lancet 2021;398:10311:1581-1592
Efficacy and Safety of NVX-CoV2373 in Adults in the United States and Mexico.	The New England journal of medicine 2022;386:6:531-543
Efficacy and safety of the CVnCoV SARS-CoV-2 mRNA vaccine candidate in ten countries in Europe and Latin America (HERALD): a randomised, observer-blinded, placebo-controlled, phase 2b/3 trial.	The Lancet. Infectious diseases 2022;22:3:329-340
Efficacy and Safety of the mRNA-1273 SARS-CoV-2 Vaccine.	The New England journal of medicine 2021;384:5:403-416
Efficacy and Safety of Therapeutic-Dose Heparin vs Standard Prophylactic or Intermediate-Dose Heparins for Thromboprophylaxis in High-risk Hospitalized Patients With COVID-19: The HEP-COVID Randomized Clinical Trial.	JAMA internal medicine 2021;181:12:1612-1620
Efficacy and safety of voclosporin versus placebo for lupus nephritis (AURORA 1): a double-blind, randomised, multicentre, placebo-controlled, phase 3 trial.	Lancet 2021;397:10289:2070-2080
Efficacy and Toxicity of Pegaspargase and Calaspargase Pegol in Childhood Acute Lymphoblastic Leukemia: Results of DFCI 11-001.	Journal of clinical oncology 2021;39:31:3496-3505
Efficacy of a Cell-Culture-Derived Quadrivalent Influenza Vaccine in Children.	The New England journal of medicine 2021;385:16:1485-

	1495
Efficacy of a Combination of Conservative Therapies vs an Education Comparator on Clinical Outcomes in Thumb Base Osteoarthritis: A Randomized Clinical Trial.	JAMA internal medicine 2021;181:4:429-438
Efficacy of Acupuncture for Chronic Prostatitis/Chronic Pelvic Pain Syndrome : A Randomized Trial.	Annals of internal medicine 2021;174:10:1357-1366
Efficacy of Blended Collaborative Care for Patients With Heart Failure and Comorbid Depression: A Randomized Clinical Trial.	JAMA internal medicine 2021;181:10:1369-1380
Efficacy of ChAdOx1 nCoV-19 (AZD1222) vaccine against SARS-CoV-2 variant of concern 202012/01 (B.1.1.7): an exploratory analysis of a randomised controlled trial.	Lancet 2021;397:10282:1351-1362
Efficacy of NVX-CoV2373 Covid-19 Vaccine against the B.1.351 Variant.	The New England journal of medicine 2021;384:20:1899-1909
Efficacy of the ChAdOx1 nCoV-19 Covid-19 Vaccine against the B.1.351 Variant.	The New England journal of medicine 2021;384:20:1885-1898
Efficacy of the mRNA-1273 SARS-CoV-2 Vaccine at Completion of Blinded Phase.	The New England journal of medicine 2021;385:19:1774-1785
Efficacy of Wolbachia-Infected Mosquito Deployments for the Control of Dengue.	The New England journal of medicine 2021;384:23:2177-2186
Efficacy, immunogenicity, and safety of a quadrivalent HPV vaccine in men: results of an open-label, long-term extension of a randomised, placebo-controlled, phase 3 trial.	The Lancet. Infectious diseases 2022;22:3:413-425

Efficacy, Safety, and Correlative Biomarkers of Toripalimab in Previously Treated Recurrent or Metastatic Nasopharyngeal Carcinoma: A Phase II Clinical Trial (POLARIS-02).	Journal of clinical oncology 2021;39:7:704-712
Efficacy, safety, and lot-to-lot immunogenicity of an inactivated SARS-CoV-2 vaccine (BBV152): interim results of a randomised, double-blind, controlled, phase 3 trial.	Lancet 2021;398:10317:2173-2184
Endoscopic surgery compared with intensity-modulated radiotherapy in resectable locally recurrent nasopharyngeal carcinoma: a multicentre, open-label, randomised, controlled, phase 3 trial.	The Lancet. Oncology 2021;22:3:381-390
Endovascular Therapy for Stroke Due to Basilar-Artery Occlusion.	The New England journal of medicine 2021;384:20:1910-1920
Enfortumab Vedotin in Previously Treated Advanced Urothelial Carcinoma.	The New England journal of medicine 2021;384:12:1125-1135
Evaluation of geriatric assessment and management on the toxic effects of cancer treatment (GAP70+): a cluster-randomised study.	Lancet 2021;398:10314:1894-1904
Evaluation of mRNA-1273 SARS-CoV-2 Vaccine in Adolescents.	The New England journal of medicine 2021;385:24:2241-2251
Evaluation of the Effects of Remdesivir and Hydroxychloroquine on Viral Clearance in COVID-19 : A Randomized Trial.	Annals of internal medicine 2021;174:9:1261-1269
Evaluation of Time-Limited Trials Among Critically Ill Patients With Advanced Medical Illnesses and Reduction of Nonbeneficial ICU Treatments.	JAMA internal medicine 2021;181:6:786-794
Excellent Outcomes With Reduced Frequency of Vincristine and Dexamethasone Pulses in Standard-Risk B-Lymphoblastic Leukemia: Results From Children's Oncology Group AALL0932.	Journal of clinical oncology 2021;39:13:1437-1447

Exercise versus usual care after non-reconstructive breast cancer surgery (UK PROSPER): multicentre randomised controlled trial and economic evaluation.	BMJ 2021;375:e066542
Extended therapy with letrozole as adjuvant treatment of postmenopausal patients with early-stage breast cancer: a multicentre, open-label, randomised, phase 3 trial.	The Lancet. Oncology 2021;22:10:1458-1467
Final efficacy analysis, interim safety analysis, and immunogenicity of a single dose of recombinant novel coronavirus vaccine (adenovirus type 5 vector) in adults 18 years and older: an international, multicentre, randomised, double-blinded, placebo-controlled phase 3 trial.	Lancet 2022;399:10321:237-248
Final Overall Survival Analysis of the TOURMALINE-MM1 Phase III Trial of Ixazomib, Lenalidomide, and Dexamethasone in Patients With Relapsed or Refractory Multiple Myeloma.	Journal of clinical oncology 2021;39:22:2430-2442
Final Report of a Trial of Intensive versus Standard Blood-Pressure Control.	The New England journal of medicine 2021;384:20:1921-1930
First-line nivolumab plus chemotherapy versus chemotherapy alone for advanced gastric, gastro-oesophageal junction, and oesophageal adenocarcinoma (CheckMate 649): a randomised, open-label, phase 3 trial.	Lancet 2021;398:10294:27-40
First-line nivolumab plus ipilimumab combined with two cycles of chemotherapy in patients with non-small-cell lung cancer (CheckMate 9LA): an international, randomised, open-label, phase 3 trial.	The Lancet. Oncology 2021;22:2:198-211
First-line nivolumab plus ipilimumab in unresectable malignant pleural mesothelioma (CheckMate 743): a multicentre, randomised, open-label, phase 3 trial.	Lancet 2021;397:10272:375-386
Five-Year Outcomes From the Randomized, Phase III Trials CheckMate 017 and 057: Nivolumab Versus Docetaxel in Previously Treated Non-Small-Cell Lung Cancer.	Journal of clinical oncology 2021;39:7:723-733
Five-Year Outcomes With Pembrolizumab Versus Chemotherapy for Metastatic Non-Small-Cell Lung Cancer With PD-L1 Tumor Proportion Score <U+2265> 50.	Journal of clinical oncology 2021;39:21:2339-2349

Focal Boost to the Intraprostatic Tumor in External Beam Radiotherapy for Patients With Localized Prostate Cancer: Results From the FLAME Randomized Phase III Trial.	Journal of clinical oncology 2021;39:7:787-796
Food Insecurity and Weight Loss in an Underserved Primary Care Population: A Post Hoc Analysis of a Cluster Randomized Trial.	Annals of internal medicine 2021;174:7:1032-1034
Fractional Flow Reserve-Guided PCI as Compared with Coronary Bypass Surgery.	The New England journal of medicine 2022;386:2:128-137
Gastric Bypass Versus Sleeve Gastrectomy in Type 2 Diabetes: Effects on Hepatic Steatosis and Fibrosis : A Randomized Controlled Trial.	Annals of internal medicine 2022;175:1:74-83
Gefitinib Versus Vinorelbine Plus Cisplatin as Adjuvant Treatment for Stage II-IIIA (N1-N2) EGFR-Mutant NSCLC: Final Overall Survival Analysis of CTONG1104 Phase III Trial.	Journal of clinical oncology 2021;39:7:713-722
Gemcitabine Plus Cisplatin Versus Fluorouracil Plus Cisplatin as First-Line Therapy for Recurrent or Metastatic Nasopharyngeal Carcinoma: Final Overall Survival Analysis of GEM20110714 Phase III Study.	Journal of clinical oncology 2021;39:29:3273-3282
Gemcitabine with or without ramucirumab as second-line treatment for malignant pleural mesothelioma (RAMES): a randomised, double-blind, placebo-controlled, phase 2 trial.	The Lancet. Oncology 2021;22:10:1438-1447
Gonadal Function Recovery in Patients With Advanced Hodgkin Lymphoma Treated With a PET-Adapted Regimen: Prospective Analysis of a Randomized Phase III Trial (AHL2011).	Journal of clinical oncology 2021;39:29:3251-3260
Haemodynamic-guided management of heart failure (GUIDE-HF): a randomised controlled trial.	Lancet 2021;398:10304:991-1001
Health-Related Quality of Life in Metastatic, Hormone-Sensitive Prostate Cancer: ENZAMET (ANZUP 1304), an International, Randomized Phase III Trial Led by ANZUP.	Journal of clinical oncology 2022;40:8:837-846
Health-related quality of life in patients with microsatellite instability-high or mismatch repair	The Lancet. Oncology

deficient metastatic colorectal cancer treated with first-line pembrolizumab versus chemotherapy (KEYNOTE-177): an open-label, randomised, phase 3 trial.	2021;22:5:665-677
Health-Related Quality of Life in Transplant-Ineligible Patients With Newly Diagnosed Multiple Myeloma: Findings From the Phase III MAIA Trial.	Journal of clinical oncology 2021;39:3:227-237
Health-Related Quality of Life With Trastuzumab Monotherapy Versus Trastuzumab Plus Standard Chemotherapy as Adjuvant Therapy in Older Patients With HER2-Positive Breast Cancer.	Journal of clinical oncology 2021;39:22:2452-2462
Healthy Weight Loss Maintenance with Exercise, Liraglutide, or Both Combined.	The New England journal of medicine 2021;384:18:1719-1730
Hepatectomy Followed by mFOLFOX6 Versus Hepatectomy Alone for Liver-Only Metastatic Colorectal Cancer (JCOG0603): A Phase II or III Randomized Controlled Trial.	Journal of clinical oncology 2021;39:34:3789-3799
Hepatic Arterial Infusion of Oxaliplatin, Fluorouracil, and Leucovorin Versus Transarterial Chemoembolization for Large Hepatocellular Carcinoma: A Randomized Phase III Trial.	Journal of clinical oncology 2022;40:2:150-160
High-dose versus standard-dose twice-daily thoracic radiotherapy for patients with limited stage small-cell lung cancer: an open-label, randomised, phase 2 trial.	The Lancet. Oncology 2021;22:3:321-331
Home and Online Management and Evaluation of Blood Pressure (HOME BP) using a digital intervention in poorly controlled hypertension: randomised controlled trial.	BMJ 2021;372:m4858
Hydroxychloroquine as Postexposure Prophylaxis to Prevent Severe Acute Respiratory Syndrome Coronavirus 2 Infection : A Randomized Trial.	Annals of internal medicine 2021;174:3:344-352
Hypothermic Machine Perfusion in Liver Transplantation - A Randomized Trial.	The New England journal of medicine 2021;384:15:1391-1401
Ibrutinib Plus Rituximab Versus Placebo Plus Rituximab for Waldenstrom's Macroglobulinemia: Final	Journal of clinical oncology

Analysis From the Randomized Phase III iNNOVATE Study.	2022;40:1:52-62
Ibrutinib Plus Venetoclax for First-Line Treatment of Chronic Lymphocytic Leukemia: Primary Analysis Results From the Minimal Residual Disease Cohort of the Randomized Phase II CAPTIVATE Study.	Journal of clinical oncology 2021;39:34:3853-3865
Immediate "Kangaroo Mother Care" and Survival of Infants with Low Birth Weight.	The New England journal of medicine 2021;384:21:2028-2038
Immediate versus Postponed Intervention for Infected Necrotizing Pancreatitis.	The New England journal of medicine 2021;385:15:1372-1381
Immunogenicity and reactogenicity of BNT162b2 booster in ChAdOx1-S-primed participants (CombiVacS): a multicentre, open-label, randomised, controlled, phase 2 trial.	Lancet 2021;398:10295:121-130
Immunogenicity and safety of a tri-antigenic versus a mono-antigenic hepatitis B vaccine in adults (PROTECT): a randomised, double-blind, phase 3 trial.	The Lancet. Infectious diseases 2021;21:9:1271-1281
Immunogenicity and safety of BNT162b2 mRNA vaccine booster in actively treated patients with cancer.	The Lancet. Oncology 2022;23:2:193-195
Immunogenicity of alternative ten-valent pneumococcal conjugate vaccine schedules in infants in Ho Chi Minh City, Vietnam: results from a single-blind, parallel-group, open-label, randomised, controlled trial.	The Lancet. Infectious diseases 2021;21:10:1415-1428
Immunogenicity of the Ad26.COV2.S Vaccine for COVID-19.	JAMA 2021;325:15:1535-1544
Immunogenicity of the UK group B meningococcal vaccine (4CMenB) schedule against groups B and	The Lancet. Infectious

C meningococcal strains (Sched3): outcomes of a multicentre, open-label, randomised controlled trial.	diseases 2021;21:5:688-696
Impact and cost-effectiveness of a lethal house lure against malaria transmission in central Cote d'Ivoire: a two-arm, cluster-randomised controlled trial.	Lancet 2021;397:10276:805-815
Impact of Pembrolizumab Versus Chemotherapy as Second-Line Therapy for Advanced Esophageal Cancer on Health-Related Quality of Life in KEYNOTE-181.	Journal of clinical oncology 2022;40:4:382-391
Inhaled and intranasal ciclesonide for the treatment of covid-19 in adult outpatients: CONTAIN phase II randomised controlled trial.	BMJ 2021;375:e068060
Inhaled Treprostinil in Pulmonary Hypertension Due to Interstitial Lung Disease.	The New England journal of medicine 2021;384:4:325-334
Inhibition of WEE1 Is Effective in TP53- and RAS-Mutant Metastatic Colorectal Cancer: A Randomized Trial (FOCUS4-C) Comparing Adavosertib (AZD1775) With Active Monitoring.	Journal of clinical oncology 2021;39:33:3705-3715
Initial treatment with a single pill containing quadruple combination of quarter doses of blood pressure medicines versus standard dose monotherapy in patients with hypertension (QUARTET): a phase 3, randomised, double-blind, active-controlled trial.	Lancet 2021;398:10305:1043-1052
Interim Results of a Phase 1-2a Trial of Ad26.COV2.S Covid-19 Vaccine.	The New England journal of medicine 2021;384:19:1824-1835
Intranasal Oxytocin in Children and Adolescents with Autism Spectrum Disorder.	The New England journal of medicine 2021;385:16:1462-1473
Ipatasertib plus abiraterone and prednisolone in metastatic castration-resistant prostate cancer (IPATential150): a multicentre, randomised, double-blind, phase 3 trial.	Lancet 2021;398:10295:131-142

Is Comprehensive Geriatric Assessment Admission Avoidance Hospital at Home an Alternative to Hospital Admission for Older Persons? : A Randomized Trial.	Annals of internal medicine 2021;174:7:889-898
Isatuximab, carfilzomib, and dexamethasone in relapsed multiple myeloma (IKEMA): a multicentre, open-label, randomised phase 3 trial.	Lancet 2021;397:10292:2361-2371
Laparoscopic Versus Open Gastrectomy for Gastric Cancer (LOGICA): A Multicenter Randomized Clinical Trial.	Journal of clinical oncology 2021;39:9:978-989
Late Toxicity After Adjuvant Conventional Radiation Versus Image-Guided Intensity-Modulated Radiotherapy for Cervical Cancer (PARCER): A Randomized Controlled Trial.	Journal of clinical oncology 2021;39:33:3682-3692
Left Atrial Appendage Occlusion during Cardiac Surgery to Prevent Stroke.	The New England journal of medicine 2021;384:22:2081-2091
Lenalidomide-Epoetin Alfa Versus Lenalidomide Monotherapy in Myelodysplastic Syndromes Refractory to Recombinant Erythropoietin.	Journal of clinical oncology 2021;39:9:1001-1009
Lenvatinib in Patients With Advanced Grade 1/2 Pancreatic and Gastrointestinal Neuroendocrine Tumors: Results of the Phase II TALENT Trial (GETNE1509).	Journal of clinical oncology 2021;39:20:2304-2312
Levonorgestrel vs. Copper Intrauterine Devices for Emergency Contraception.	The New England journal of medicine 2021;384:4:335-344
Linked Color Imaging Focused on Neoplasm Detection in the Upper Gastrointestinal Tract : A Randomized Trial.	Annals of internal medicine 2021;174:1:18-24
Liposomal irinotecan plus fluorouracil and leucovorin versus fluorouracil and leucovorin for metastatic biliary tract cancer after progression on gemcitabine plus cisplatin (NIFTY): a multicentre, open-label, randomised, phase 2b study.	The Lancet. Oncology 2021;22:11:1560-1572

Live Birth with or without Preimplantation Genetic Testing for Aneuploidy.	The New England journal of medicine 2021;385:22:2047-2058
Long-Term Oncologic Outcomes After Laparoscopic Versus Open Resection for Colorectal Liver Metastases : A Randomized Trial.	Annals of internal medicine 2021;174:2:175-182
Long-term outcomes of patients with active melanoma brain metastases treated with combination nivolumab plus ipilimumab (CheckMate 204): final results of an open-label, multicentre, phase 2 study.	The Lancet. Oncology 2021;22:12:1692-1704
Lower or Higher Oxygenation Targets for Acute Hypoxemic Respiratory Failure.	The New England journal of medicine 2021;384:14:1301-1311
Lumasiran, an RNAi Therapeutic for Primary Hyperoxaluria Type 1.	The New England journal of medicine 2021;384:13:1216-1226
Lutetium-177-PSMA-617 for Metastatic Castration-Resistant Prostate Cancer.	The New England journal of medicine 2021;385:12:1091-1103
Maintenance olaparib for patients with newly diagnosed advanced ovarian cancer and a BRCA mutation (SOLO1/GOG 3004): 5-year follow-up of a randomised, double-blind, placebo-controlled, phase 3 trial.	The Lancet. Oncology 2021;22:12:1721-1731
Maintenance or Discontinuation of Antidepressants in Primary Care.	The New England journal of medicine 2021;385:14:1257-1267

Maintenance with daratumumab or observation following treatment with bortezomib, thalidomide, and dexamethasone with or without daratumumab and autologous stem-cell transplant in patients with newly diagnosed multiple myeloma (CASSIOPEIA): an open-label, randomised, phase 3 trial.	The Lancet. Oncology 2021;22:10:1378-1390
Mass drug administration for the acceleration of malaria elimination in a region of Myanmar with artemisinin-resistant falciparum malaria: a cluster-randomised trial.	The Lancet. Infectious diseases 2021;21:11:1579-1589
Mass drug administration of ivermectin and dihydroartemisinin-piperaquine against malaria in settings with high coverage of standard control interventions: a cluster-randomised controlled trial in The Gambia.	The Lancet. Infectious diseases 2022;22:4:519-528
Mavacamten for treatment of symptomatic obstructive hypertrophic cardiomyopathy (EXPLORER-HCM): health status analysis of a randomised, double-blind, placebo-controlled, phase 3 trial.	Lancet 2021;397:10293:2467-2475
Metronomic capecitabine as adjuvant therapy in locoregionally advanced nasopharyngeal carcinoma: a multicentre, open-label, parallel-group, randomised, controlled, phase 3 trial.	Lancet 2021;398:10297:303-313
Milrinone as Compared with Dobutamine in the Treatment of Cardiogenic Shock.	The New England journal of medicine 2021;385:6:516-525
Minimal Residual Disease Dynamics after Venetoclax-Obinutuzumab Treatment: Extended Off-Treatment Follow-up From the Randomized CLL14 Study.	Journal of clinical oncology 2021;39:36:4049-4060
Modified FOLFIRINOX Versus CISGEM Chemotherapy for Patients With Advanced Biliary Tract Cancer (PRODIGE 38 AMEBICA): A Randomized Phase II Study.	Journal of clinical oncology 2022;40:3:262-271
Molnupiravir for Oral Treatment of Covid-19 in Nonhospitalized Patients.	The New England journal of medicine 2022;386:6:509-520

MRI-Targeted or Standard Biopsy in Prostate Cancer Screening.	The New England journal of medicine 2021;385:10:908-920
Multicomponent Strategy with Decentralized Molecular Testing for Tuberculosis.	The New England journal of medicine 2021;385:26:2441-2450
Multifactorial falls prevention programme compared with usual care in UK care homes for older people: multicentre cluster randomised controlled trial with economic evaluation.	BMJ 2021;375:e066991
Multivessel PCI Guided by FFR or Angiography for Myocardial Infarction.	The New England journal of medicine 2021;385:4:297-308
Muscarinic Cholinergic Receptor Agonist and Peripheral Antagonist for Schizophrenia.	The New England journal of medicine 2021;384:8:717-726
Mycophenolate Mofetil for First-Line Treatment of Immune Thrombocytopenia.	The New England journal of medicine 2021;385:10:885-895
nab-Sirolimus for Patients With Malignant Perivascular Epithelioid Cell Tumors.	Journal of clinical oncology 2021;39:33:3660-3670
Neck Dissections Based on Sentinel Lymph Node Navigation Versus Elective Neck Dissections in Early Oral Cancers: A Randomized, Multicenter, and Noninferiority Trial.	Journal of clinical oncology 2021;39:18:2025-2036
Neoadjuvant chemotherapy with FOLFIRINOX and preoperative chemoradiotherapy for patients with locally advanced rectal cancer (UNICANCER-PRODIGE 23): a multicentre, randomised, open-label,	The Lancet. Oncology 2021;22:5:702-715

phase 3 trial.	
Neoadjuvant durvalumab with or without stereotactic body radiotherapy in patients with early-stage non-small-cell lung cancer: a single-centre, randomised phase 2 trial.	The Lancet. Oncology 2021;22:6:824-835
Neutralization of SARS-CoV-2 Variants in Transplant Recipients After Two and Three Doses of mRNA-1273 Vaccine : Secondary Analysis of a Randomized Trial.	Annals of internal medicine 2022;175:2:226-233
Nivolumab and Ipilimumab as Maintenance Therapy in Extensive-Disease Small-Cell Lung Cancer: CheckMate 451.	Journal of clinical oncology 2021;39:12:1349-1359
Nivolumab plus Cabozantinib versus Sunitinib for Advanced Renal-Cell Carcinoma.	The New England journal of medicine 2021;384:9:829-841
Nivolumab Versus Gemcitabine or Pegylated Liposomal Doxorubicin for Patients With Platinum-Resistant Ovarian Cancer: Open-Label, Randomized Trial in Japan (NINJA).	Journal of clinical oncology 2021;39:33:3671-3681
Nivolumab versus placebo in patients with relapsed malignant mesothelioma (CONFIRM): a multicentre, double-blind, randomised, phase 3 trial.	The Lancet. Oncology 2021;22:11:1530-1540
Nivolumab versus sorafenib in advanced hepatocellular carcinoma (CheckMate 459): a randomised, multicentre, open-label, phase 3 trial.	The Lancet. Oncology 2022;23:1:77-90
Occipital nerve stimulation for chronic cluster headache.	The Lancet. Neurology 2021;20:7:498-499
Olaparib tablets as maintenance therapy in patients with platinum-sensitive relapsed ovarian cancer and a BRCA1/2 mutation (SOLO2/ENGOT-Ov21): a final analysis of a double-blind, randomised, placebo-controlled, phase 3 trial.	The Lancet. Oncology 2021;22:5:620-631
Once-weekly cagrilintide for weight management in people with overweight and obesity: a multicentre, randomised, double-blind, placebo-controlled and active-controlled, dose-finding phase 2	Lancet 2021;398:10317:2160-2172

trial.	
Once-Weekly Semaglutide in Adults with Overweight or Obesity.	The New England journal of medicine 2021;384:11:989
Open-Label Phase II Prospective, Randomized, Controlled Study of Romyelocel-L Myeloid Progenitor Cells to Reduce Infection During Induction Chemotherapy for Acute Myeloid Leukemia.	Journal of clinical oncology 2021;39:29:3261-3272
Optimizing Therapy to Prevent Avoidable Hospital Admissions in Multimorbid Older Adults (OPERAM): cluster randomised controlled trial.	BMJ 2021;374:n1585
Oral rimegepant for preventive treatment of migraine: a phase 2/3, randomised, double-blind, placebo-controlled trial.	Lancet 2021;397:10268:51-60
Outcomes by Clinical and Molecular Features in Children With Medulloblastoma Treated With Risk-Adapted Therapy: Results of an International Phase III Trial (SJMB03).	Journal of clinical oncology 2021;39:7:822-835
Overall Survival Benefit with Tebentafusp in Metastatic Uveal Melanoma.	The New England journal of medicine 2021;385:13:1196-1206
Overall survival in patients with hormone receptor-positive, HER2-negative, advanced or metastatic breast cancer treated with a cyclin-dependent kinase 4/6 inhibitor plus fulvestrant: a US Food and Drug Administration pooled analysis.	The Lancet. Oncology 2021;22:11:1573-1581
Ozanimod as Induction and Maintenance Therapy for Ulcerative Colitis.	The New England journal of medicine 2021;385:14:1280-1291
Palbociclib for Residual High-Risk Invasive HR-Positive and HER2-Negative Early Breast Cancer-The Penelope-B Trial.	Journal of clinical oncology 2021;39:14:1518-1530
Palbociclib with adjuvant endocrine therapy in early breast cancer (PALLAS): interim analysis of a	The Lancet. Oncology

multicentre, open-label, randomised, phase 3 study.	2021;22:2:212-222
Pan-AKT Inhibitor Capivasertib With Docetaxel and Prednisolone in Metastatic Castration-Resistant Prostate Cancer: A Randomized, Placebo-Controlled Phase II Trial (ProCAID).	Journal of clinical oncology 2021;39:3:190-201
Panitumumab Plus Fluorouracil and Folinic Acid Versus Fluorouracil and Folinic Acid Alone as Maintenance Therapy in RAS Wild-Type Metastatic Colorectal Cancer: The Randomized PANAMA Trial (AIO KRK 0212).	Journal of clinical oncology 2022;40:1:72-82
Patient-centred outcomes and effect of disease progression on health status in patients with newly diagnosed advanced ovarian cancer and a BRCA mutation receiving maintenance olaparib or placebo (SOLO1): a randomised, phase 3 trial.	The Lancet. Oncology 2021;22:5:632-642
Patient-reported outcomes with atezolizumab plus bevacizumab versus sorafenib in patients with unresectable hepatocellular carcinoma (IMbrave150): an open-label, randomised, phase 3 trial.	The Lancet. Oncology 2021;22:7:991-1001
Pegcetacoplan versus Eculizumab in Paroxysmal Nocturnal Hemoglobinuria.	The New England journal of medicine 2021;384:11:1028-1037
Pembrolizumab for Persistent, Recurrent, or Metastatic Cervical Cancer.	The New England journal of medicine 2021;385:20:1856-1867
Pembrolizumab plus chemotherapy versus chemotherapy alone for first-line treatment of advanced oesophageal cancer (KEYNOTE-590): a randomised, placebo-controlled, phase 3 study.	Lancet 2021;398:10302:759-771
Pembrolizumab Plus Ipilimumab Following Anti-PD-1/L1 Failure in Melanoma.	Journal of clinical oncology 2021;39:24:2647-2655
Pembrolizumab Plus Ipilimumab or Placebo for Metastatic Non-Small-Cell Lung Cancer With PD-L1 Tumor Proportion Score <U+2265> 50%: Randomized, Double-Blind Phase III KEYNOTE-598	Journal of clinical oncology 2021;39:21:2327-2338

Study.	
Pembrolizumab versus brentuximab vedotin in relapsed or refractory classical Hodgkin lymphoma (KEYNOTE-204): an interim analysis of a multicentre, randomised, open-label, phase 3 study.	The Lancet. Oncology 2021;22:4:512-524
Pembrolizumab versus investigator-choice chemotherapy for metastatic triple-negative breast cancer (KEYNOTE-119): a randomised, open-label, phase 3 trial.	The Lancet. Oncology 2021;22:4:499-511
PET-guided omission of radiotherapy in early-stage unfavourable Hodgkin lymphoma (GHSG HD17): a multicentre, open-label, randomised, phase 3 trial.	The Lancet. Oncology 2021;22:2:223-234
Phase 3 Safety and Efficacy of AZD1222 (ChAdOx1 nCoV-19) Covid-19 Vaccine.	The New England journal of medicine 2021;385:25:2348-2360
Phase 3 Trial of Interleukin-1 Trap Rilonacept in Recurrent Pericarditis.	The New England journal of medicine 2021;384:1:31-41
Phase 3 Trials of Tapinarof Cream for Plaque Psoriasis.	The New England journal of medicine 2021;385:24:2219-2229
Phase 3 Trials of Tirbanibulin Ointment for Actinic Keratosis.	The New England journal of medicine 2021;384:6:512-520
Phase II Randomized Study of CMB305 and Atezolizumab Compared With Atezolizumab Alone in Soft-Tissue Sarcomas Expressing NY-ESO-1.	Journal of clinical oncology 2022;40:12:1291-1300
Phase II Trial of Costimulation Blockade With Abatacept for Prevention of Acute GVHD.	Journal of clinical oncology 2021;39:17:1865-1877
Phase III Randomized Controlled Trial of eRAPID: eHealth Intervention During Chemotherapy.	Journal of clinical oncology

	2021;39:7:734-747
Phase III Study to Evaluate Efficacy and Safety of Andecaliximab With mFOLFOX6 as First-Line Treatment in Patients With Advanced Gastric or GEJ Adenocarcinoma (GAMMA-1).	Journal of clinical oncology 2021;39:9:990-1000
Phase III Trial Adding Vincristine-Topotecan-Cyclophosphamide to the Initial Treatment of Patients With Nonmetastatic Ewing Sarcoma: A Children's Oncology Group Report.	Journal of clinical oncology 2021;39:36:4029-4038
Phase III Trial of Avelumab Maintenance After First-Line Induction Chemotherapy Versus Continuation of Chemotherapy in Patients With Gastric Cancers: Results From JAVELIN Gastric 100.	Journal of clinical oncology 2021;39:9:966-977
Phase III, Randomized, Placebo-Controlled Trial of CC-486 (Oral Azacitidine) in Patients With Lower-Risk Myelodysplastic Syndromes.	Journal of clinical oncology 2021;39:13:1426-1436
Physical Rehabilitation for Older Patients Hospitalized for Heart Failure.	The New England journal of medicine 2021;385:3:203-216
Physical Therapy Referral From Primary Care for Acute Back Pain With Sciatica : A Randomized Controlled Trial.	Annals of internal medicine 2021;174:1:8-17
Placental growth factor in assessment of women with suspected pre-eclampsia to reduce maternal morbidity: a stepped wedge cluster randomised control trial (PARROT Ireland).	BMJ 2021;374:n1857
Polatuzumab Vedotin in Previously Untreated Diffuse Large B-Cell Lymphoma.	The New England journal of medicine 2022;386:4:351-363
Polypill with or without Aspirin in Persons without Cardiovascular Disease.	The New England journal of medicine 2021;384:3:216-228
Posaconazole versus voriconazole for primary treatment of invasive aspergillosis: a phase 3,	Lancet 2021;397:10273:499-

randomised, controlled, non-inferiority trial.	509
Postoperative low molecular weight heparin bridging treatment for patients at high risk of arterial thromboembolism (PERIOP2): double blind randomised controlled trial.	BMJ 2021;373:n1205
Postoperative radiotherapy versus no postoperative radiotherapy in patients with completely resected non-small-cell lung cancer and proven mediastinal N2 involvement (Lung ART): an open-label, randomised, phase 3 trial.	The Lancet. Oncology 2022;23:1:104-114
Pre- and Postoperative Capecitabine Without or With Oxaliplatin in Locally Advanced Rectal Cancer: PETACC 6 Trial by EORTC GITCG and ROG, AIO, AGITG, BGDO, and FFCD.	Journal of clinical oncology 2021;39:1:17-29
Preventing Hospital Readmission for Patients With Comorbid Substance Use Disorder : A Randomized Trial.	Annals of internal medicine 2021;174:7:899-909
Prevention of influenza during mismatched seasons in older adults with an MF59-adjuvanted quadrivalent influenza vaccine: a randomised, controlled, multicentre, phase 3 efficacy study.	The Lancet. Infectious diseases 2021;21:7:1027-1037
Primary Tumor Resection Plus Chemotherapy Versus Chemotherapy Alone for Colorectal Cancer Patients With Asymptomatic, Synchronous Unresectable Metastases (JCOG1007; iPACS): A Randomized Clinical Trial.	Journal of clinical oncology 2021;39:10:1098-1107
Procalcitonin and lung ultrasonography point-of-care testing to determine antibiotic prescription in patients with lower respiratory tract infection in primary care: pragmatic cluster randomised trial.	BMJ 2021;374:n2132
PRODIGY: A Phase III Study of Neoadjuvant Docetaxel, Oxaliplatin, and S-1 Plus Surgery and Adjuvant S-1 Versus Surgery and Adjuvant S-1 for Resectable Advanced Gastric Cancer.	Journal of clinical oncology 2021;39:26:2903-2913
Progressive exercise compared with best practice advice, with or without corticosteroid injection, for the treatment of patients with rotator cuff disorders (GRASP): a multicentre, pragmatic, 2<U+2009>×<U+2009>2 factorial, randomised controlled trial.	Lancet 2021;398:10298:416-428

Prospective Phase II Open-Label Randomized Controlled Trial to Compare Mandibular Preservation in Upfront Surgery With Neoadjuvant Chemotherapy Followed by Surgery in Operable Oral Cavity Cancer.	Journal of clinical oncology 2022;40:3:272-281
Prostate Radiotherapy With Adjuvant Androgen Deprivation Therapy (ADT) Improves Metastasis-Free Survival Compared to Neoadjuvant ADT: An Individual Patient Meta-Analysis.	Journal of clinical oncology 2021;39:2:136-144
Prostate-Only Versus Whole-Pelvic Radiation Therapy in High-Risk and Very High-Risk Prostate Cancer (POP-RT): Outcomes From Phase III Randomized Controlled Trial.	Journal of clinical oncology 2021;39:11:1234-1242
Quality of Life in Men With Prostate Cancer Randomly Allocated to Receive Docetaxel or Abiraterone in the STAMPEDE Trial.	Journal of clinical oncology 2022;40:8:825-836
Radiation and Androgen Deprivation Therapy With or Without Docetaxel in the Management of Nonmetastatic Unfavorable-Risk Prostate Cancer: A Prospective Randomized Trial.	Journal of clinical oncology 2021;39:26:2938-2947
Radical Cystectomy Against Intravesical BCG for High-Risk High-Grade Nonmuscle Invasive Bladder Cancer: Results From the Randomized Controlled BRAVO-Feasibility Study.	Journal of clinical oncology 2021;39:3:202-214
Radioembolization With Chemotherapy for Colorectal Liver Metastases: A Randomized, Open-Label, International, Multicenter, Phase III Trial.	Journal of clinical oncology 2021;39:35:3897-3907
Randomized Controlled Phase II Evaluation of Two Dose Levels of Bupropion Versus Placebo for Sexual Desire in Female Cancer Survivors: NRG-CC004.	Journal of clinical oncology 2022;40:4:324-334
Randomized Phase II Study of Bevacizumab in Combination With Carboplatin Plus Paclitaxel in Patients With Previously Untreated Advanced Mucosal Melanoma.	Journal of clinical oncology 2021;39:8:881-889
Randomized Phase II Study of PET Response-Adapted Combined Modality Therapy for Esophageal Cancer: Mature Results of the CALGB 80803 (Alliance) Trial.	Journal of clinical oncology 2021;39:25:2803-2815
Randomized Phase II Trial Evaluating Two Sequential Treatments in First Line of Metastatic Pancreatic Cancer: Results of the PANOPTIMOX-PRODIGE 35 Trial.	Journal of clinical oncology 2021;39:29:3242-3250

Randomized Phase III BMT CTN Trial of Calcineurin Inhibitor-Free Chronic Graft-Versus-Host Disease Interventions in Myeloablative Hematopoietic Cell Transplantation for Hematologic Malignancies.	Journal of clinical oncology 2022;40:4:356-368
Randomized Phase III Postoperative Trial of Platinum-Based Chemotherapy Versus Capecitabine in Patients With Residual Triple-Negative Breast Cancer Following Neoadjuvant Chemotherapy: ECOG-ACRIN EA1131.	Journal of clinical oncology 2021;39:23:2539-2551
Randomized Phase III Study of FOLFOX Alone or With Pegilodecakin as Second-Line Therapy in Patients With Metastatic Pancreatic Cancer That Progressed After Gemcitabine (SEQUOIA).	Journal of clinical oncology 2021;39:10:1108-1118
Randomized Phase III Study of Gefitinib Versus Cisplatin Plus Vinorelbine for Patients With Resected Stage II-IIIA Non-Small-Cell Lung Cancer With EGFR Mutation (IMPACT).	Journal of clinical oncology 2022;40:3:231-241
Randomized Phase III Trial Evaluating Radiation Following Surgical Excision for Good-Risk Ductal Carcinoma In Situ: Long-Term Report From NRG Oncology/RTOG 9804.	Journal of clinical oncology 2021;39:32:3574-3582
Randomized Phase III Trial of Gemcitabine and Cisplatin With Bevacizumab or Placebo in Patients With Advanced Urothelial Carcinoma: Results of CALGB 90601 (Alliance).	Journal of clinical oncology 2021;39:22:2486-2496
Randomized Phase III Trial of Prophylactic Cranial Irradiation With or Without Hippocampal Avoidance for Small-Cell Lung Cancer (PREMER): A GICOR-GOECP-SEOR Study.	Journal of clinical oncology 2021;39:28:3118-3127
Randomized Study on Dose Escalation in Definitive Chemoradiation for Patients With Locally Advanced Esophageal Cancer (ARTDECO Study).	Journal of clinical oncology 2021;39:25:2816-2824
Randomized Trial of a Vaccine Regimen to Prevent Chronic HCV Infection.	The New England journal of medicine 2021;384:6:541-549
Randomized Trial of Cytoreductive Surgery for Relapsed Ovarian Cancer.	The New England journal of medicine 2021;385:23:2123-

	2131
Randomized Trial of Fetal Surgery for Moderate Left Diaphragmatic Hernia.	The New England journal of medicine 2021;385:2:119-129
Randomized Trial of Irinotecan and Cetuximab With or Without Vemurafenib in BRAF-Mutant Metastatic Colorectal Cancer (SWOG S1406).	Journal of clinical oncology 2021;39:4:285-294
Randomized, Double-Blind, Phase III Study of Fosnetupitant Versus Fosaprepitant for Prevention of Highly Emetogenic Chemotherapy-Induced Nausea and Vomiting: CONSOLE.	Journal of clinical oncology 2022;40:2:180-188
Randomized, Single-Blind, Multicenter Phase II Study of Two Doses of Imetelstat in Relapsed or Refractory Myelofibrosis.	Journal of clinical oncology 2021;39:26:2881-2892
Real time remote symptom monitoring during chemotherapy for cancer: European multicentre randomised controlled trial (eSMART).	BMJ 2021;374:n1647
REGEN-COV Antibody Combination and Outcomes in Outpatients with Covid-19.	The New England journal of medicine 2021;385:23:e81
Remote, proactive, telephone based management of toxicity in outpatients during adjuvant or neoadjuvant chemotherapy for early stage breast cancer: pragmatic, cluster randomised trial.	BMJ 2021;375:e066588
Repurposed Antiviral Drugs for Covid-19 - Interim WHO Solidarity Trial Results.	The New England journal of medicine 2021;384:6:497-511
Resistance Testing for Management of HIV Virologic Failure in Sub-Saharan Africa : An Unblinded Randomized Controlled Trial.	Annals of internal medicine 2021;174:12:1683-1692
Response-Adapted Postinduction Strategy in Patients With Advanced-Stage Follicular Lymphoma: The FOLL12 Study.	Journal of clinical oncology 2022;40:7:729-739

Responses to a Neutralizing Monoclonal Antibody for Hospitalized Patients With COVID-19 According to Baseline Antibody and Antigen Levels : A Randomized Controlled Trial.	Annals of internal medicine 2022;175:2:234-243
Risankizumab in Severe Asthma - A Phase 2a, Placebo-Controlled Trial.	The New England journal of medicine 2021;385:18:1669-1679
Risk-Based Selection of Individuals for Oral Cancer Screening.	Journal of clinical oncology 2021;39:6:663-674
Rituximab versus Mycophenolate Mofetil in Patients with Pemphigus Vulgaris.	The New England journal of medicine 2021;384:24:2295-2305
ROBUST: A Phase III Study of Lenalidomide Plus R-CHOP Versus Placebo Plus R-CHOP in Previously Untreated Patients With ABC-Type Diffuse Large B-Cell Lymphoma.	Journal of clinical oncology 2021;39:12:1317-1328
Romidepsin Plus CHOP Versus CHOP in Patients With Previously Untreated Peripheral T-Cell Lymphoma: Results of the Ro-CHOP Phase III Study (Conducted by LYSA).	Journal of clinical oncology 2022;40:3:242-251
Ruxolitinib for Glucocorticoid-Refractory Chronic Graft-versus-Host Disease.	The New England journal of medicine 2021;385:3:228-238
Sacituzumab Govitecan in Metastatic Triple-Negative Breast Cancer.	The New England journal of medicine 2021;384:16:1529-1541
Safety and Efficacy of a Typhoid Conjugate Vaccine in Malawian Children.	The New England journal of medicine 2021;385:12:1104-1115

Safety and efficacy of an rAd26 and rAd5 vector-based heterologous prime-boost COVID-19 vaccine: an interim analysis of a randomised controlled phase 3 trial in Russia.	Lancet 2021;397:10275:671-681
Safety and efficacy of avalglucosidase alfa versus alglucosidase alfa in patients with late-onset Pompe disease (COMET): a phase 3, randomised, multicentre trial.	The Lancet. Neurology 2021;20:12:1012-1026
Safety and efficacy of bexarotene in patients with relapsing-remitting multiple sclerosis (CCMR One): a randomised, double-blind, placebo-controlled, parallel-group, phase 2a study.	The Lancet. Neurology 2021;20:9:709-720
Safety and efficacy of cipaglucosidase alfa plus miglustat versus alglucosidase alfa plus placebo in late-onset Pompe disease (PROPEL): an international, randomised, double-blind, parallel-group, phase 3 trial.	The Lancet. Neurology 2021;20:12:1027-1037
Safety and efficacy of erythropoietin for the treatment of patients with optic neuritis (TONE): a randomised, double-blind, multicentre, placebo-controlled study.	The Lancet. Neurology 2021;20:12:991-1000
Safety and efficacy of losartan for the reduction of brain atrophy in clinically diagnosed Alzheimer's disease (the RADAR trial): a double-blind, randomised, placebo-controlled, phase 2 trial.	The Lancet. Neurology 2021;20:11:895-906
Safety and Efficacy of NVX-CoV2373 Covid-19 Vaccine.	The New England journal of medicine 2021;385:13:1172-1183
Safety and efficacy of occipital nerve stimulation for attack prevention in medically intractable chronic cluster headache (ICON): a randomised, double-blind, multicentre, phase 3, electrical dose-controlled trial.	The Lancet. Neurology 2021;20:7:515-525
Safety and efficacy of oral levosimendan in people with amyotrophic lateral sclerosis (the REFALS study): a randomised, double-blind, placebo-controlled phase 3 trial.	The Lancet. Neurology 2021;20:10:821-831
Safety and efficacy of pioglitazone for the delay of cognitive impairment in people at risk of Alzheimer's disease (TOMMORROW): a prognostic biomarker study and a phase 3, randomised,	The Lancet. Neurology 2021;20:7:537-547

double-blind, placebo-controlled trial.	
Safety and efficacy of prednisone versus placebo in short-term prevention of episodic cluster headache: a multicentre, double-blind, randomised controlled trial.	The Lancet. Neurology 2021;20:1:29-37
Safety and efficacy of riluzole in patients undergoing decompressive surgery for degenerative cervical myelopathy (CSM-Protect): a multicentre, double-blind, placebo-controlled, randomised, phase 3 trial.	The Lancet. Neurology 2021;20:2:98-106
Safety and Efficacy of Single-Dose Ad26.COV2.S Vaccine against Covid-19.	The New England journal of medicine 2021;384:23:2187-2201
Safety and efficacy of teriflunomide in paediatric multiple sclerosis (TERIKIDS): a multicentre, double-blind, phase 3, randomised, placebo-controlled trial.	The Lancet. Neurology 2021;20:12:1001-1011
Safety and Efficacy of the BNT162b2 mRNA Covid-19 Vaccine through 6 Months.	The New England journal of medicine 2021;385:19:1761-1773
Safety and efficacy of tilavonemab in progressive supranuclear palsy: a phase 2, randomised, placebo-controlled trial.	The Lancet. Neurology 2021;20:3:182-192
Safety and immunogenicity of a synthetic carbohydrate conjugate vaccine against Shigella flexneri 2a in healthy adult volunteers: a phase 1, dose-escalating, single-blind, randomised, placebo-controlled study.	The Lancet. Infectious diseases 2021;21:4:546-558
Safety and immunogenicity of an inactivated SARS-CoV-2 vaccine, BBV152: a double-blind, randomised, phase 1 trial.	The Lancet. Infectious diseases 2021;21:5:637-646
Safety and immunogenicity of an inactivated SARS-CoV-2 vaccine, BBV152: interim results from a double-blind, randomised, multicentre, phase 2 trial, and 3-month follow-up of a double-blind,	The Lancet. Infectious diseases 2021;21:7:950-961

randomised phase 1 trial.	
Safety and immunogenicity of ChAdOx1 nCoV-19 vaccine administered in a prime-boost regimen in young and old adults (COV002): a single-blind, randomised, controlled, phase 2/3 trial.	Lancet 2021;396:10267:1979-1993
Safety and immunogenicity of co-administered hookworm vaccine candidates Na-GST-1 and Na-APR-1 in Gabonese adults: a randomised, controlled, double-blind, phase 1 dose-escalation trial.	The Lancet. Infectious diseases 2021;21:2:275-285
Safety and immunogenicity of concomitant administration of COVID-19 vaccines (ChAdOx1 or BNT162b2) with seasonal influenza vaccines in adults in the UK (ComFluCOV): a multicentre, randomised, controlled, phase 4 trial.	Lancet 2021;398:10318:2277-2287
Safety and immunogenicity of seven COVID-19 vaccines as a third dose (booster) following two doses of ChAdOx1 nCov-19 or BNT162b2 in the UK (COV-BOOST): a blinded, multicentre, randomised, controlled, phase 2 trial.	Lancet 2021;398:10318:2258-2276
Safety and immunogenicity of S-Trimer (SCB-2019), a protein subunit vaccine candidate for COVID-19 in healthy adults: a phase 1, randomised, double-blind, placebo-controlled trial.	Lancet 2021;397:10275:682-694
Safety and immunogenicity of the two-dose heterologous Ad26.ZEBOV and MVA-BN-Filo Ebola vaccine regimen in children in Sierra Leone: a randomised, double-blind, controlled trial.	The Lancet. Infectious diseases 2022;22:1:110-122
Safety and immunogenicity of two novel type 2 oral poliovirus vaccine candidates compared with a monovalent type 2 oral poliovirus vaccine in children and infants: two clinical trials.	Lancet 2021;397:10268:27-38
Safety and immunogenicity of two novel type 2 oral poliovirus vaccine candidates compared with a monovalent type 2 oral poliovirus vaccine in healthy adults: two clinical trials.	Lancet 2021;397:10268:39-50
Safety and long-term immunogenicity of the two-dose heterologous Ad26.ZEBOV and MVA-BN-Filo Ebola vaccine regimen in adults in Sierra Leone: a combined open-label, non-randomised stage 1, and a randomised, double-blind, controlled stage 2 trial.	The Lancet. Infectious diseases 2022;22:1:97-109
Safety, efficacy, and tolerability of efgartigimod in patients with generalised myasthenia gravis	The Lancet. Neurology

(ADAPT): a multicentre, randomised, placebo-controlled, phase 3 trial.	2021;20:7:526-536
Safety, immunogenicity, and efficacy of a Clostridioides difficile toxoid vaccine candidate: a phase 3 multicentre, observer-blind, randomised, controlled trial.	The Lancet. Infectious diseases 2021;21:2:252-262
Safety, Immunogenicity, and Efficacy of the BNT162b2 Covid-19 Vaccine in Adolescents.	The New England journal of medicine 2021;385:3:239-250
Safety, tolerability, and immunogenicity of an inactivated SARS-CoV-2 vaccine (CoronaVac) in healthy adults aged 60 years and older: a randomised, double-blind, placebo-controlled, phase 1/2 clinical trial.	The Lancet. Infectious diseases 2021;21:6:803-812
Safety, tolerability, and immunogenicity of an inactivated SARS-CoV-2 vaccine (CoronaVac) in healthy children and adolescents: a double-blind, randomised, controlled, phase 1/2 clinical trial.	The Lancet. Infectious diseases 2021;21:12:1645-1653
Safety, tolerability, and immunogenicity of an inactivated SARS-CoV-2 vaccine in healthy adults aged 18-59 years: a randomised, double-blind, placebo-controlled, phase 1/2 clinical trial.	The Lancet. Infectious diseases 2021;21:2:181-192
Safety, tolerability, pharmacokinetics, and pharmacodynamics of concomitant administration of multiple doses of cagrilintide with semaglutide 2・4 mg for weight management: a randomised, controlled, phase 1b trial.	Lancet 2021;397:10286:1736-1748
Same-day SARS-CoV-2 antigen test screening in an indoor mass-gathering live music event: a randomised controlled trial.	The Lancet. Infectious diseases 2021;21:10:1365-1372
SARS-CoV-2 Neutralization with BNT162b2 Vaccine Dose 3.	The New England journal of medicine 2021;385:17:1627-

	1629
SARS-CoV-2 Neutralizing Antibody LY-CoV555 in Outpatients with Covid-19.	The New England journal of medicine 2021;384:3:229-237
Seasonal Malaria Vaccination with or without Seasonal Malaria Chemoprevention.	The New England journal of medicine 2021;385:11:1005-1017
Second intravenous immunoglobulin dose in patients with Guillain-Barre syndrome with poor prognosis (SID-GBS): a double-blind, randomised, placebo-controlled trial.	The Lancet. Neurology 2021;20:4:275-283
Secondary Cytoreduction and Carboplatin Hyperthermic Intraperitoneal Chemotherapy for Platinum-Sensitive Recurrent Ovarian Cancer: An MSK Team Ovary Phase II Study.	Journal of clinical oncology 2021;39:23:2594-2604
Secondary cytoreduction followed by chemotherapy versus chemotherapy alone in platinum-sensitive relapsed ovarian cancer (SOC-1): a multicentre, open-label, randomised, phase 3 trial.	The Lancet. Oncology 2021;22:4:439-449
Second-line FOLFOX chemotherapy versus active symptom control for advanced biliary tract cancer (ABC-06): a phase 3, open-label, randomised, controlled trial.	The Lancet. Oncology 2021;22:5:690-701
Second-Line Tisagenlecleucel or Standard Care in Aggressive B-Cell Lymphoma.	The New England journal of medicine 2022;386:7:629-639
Short Androgen Suppression and Radiation Dose Escalation in Prostate Cancer: 12-Year Results of EORTC Trial 22991 in Patients With Localized Intermediate-Risk Disease.	Journal of clinical oncology 2021;39:27:3022-3033
Short-course radiotherapy followed by chemotherapy before total mesorectal excision (TME) versus preoperative chemoradiotherapy, TME, and optional adjuvant chemotherapy in locally advanced rectal cancer (RAPIDO): a randomised, open-label, phase 3 trial.	The Lancet. Oncology 2021;22:1:29-42

Short-term outcomes of complete mesocolic excision versus D2 dissection in patients undergoing laparoscopic colectomy for right colon cancer (RELARC): a randomised, controlled, phase 3, superiority trial.	The Lancet. Oncology 2021;22:3:391-401
Sintilimab plus a bevacizumab biosimilar (IBI305) versus sorafenib in unresectable hepatocellular carcinoma (ORIENT-32): a randomised, open-label, phase 2-3 study.	The Lancet. Oncology 2021;22:7:977-990
Sotagliflozin in Patients with Diabetes and Chronic Kidney Disease.	The New England journal of medicine 2021;384:2:129-139
Sotagliflozin in Patients with Diabetes and Recent Worsening Heart Failure.	The New England journal of medicine 2021;384:2:117-128
Sotatercept for the Treatment of Pulmonary Arterial Hypertension.	The New England journal of medicine 2021;384:13:1204-1215
Standard dose raltegravir or efavirenz-based antiretroviral treatment for patients co-infected with HIV and tuberculosis (ANRS 12<U+2008>300 Reflate TB 2): an open-label, non-inferiority, randomised, phase 3 trial.	The Lancet. Infectious diseases 2021;21:6:813-822
Statin treatment and muscle symptoms: series of randomised, placebo controlled n-of-1 trials.	BMJ 2021;372:n135
Stepped Exercise Program for Patients With Knee Osteoarthritis : A Randomized Controlled Trial.	Annals of internal medicine 2021;174:3:298-307
Stereotactic body radiotherapy plus pembrolizumab and trametinib versus stereotactic body radiotherapy plus gemcitabine for locally recurrent pancreatic cancer after surgical resection: an open-label, randomised, controlled, phase 2 trial.	The Lancet. Oncology 2021;22:8:1093-1102

Stereotactic body radiotherapy versus conventional external beam radiotherapy in patients with painful spinal metastases: an open-label, multicentre, randomised, controlled, phase 2/3 trial.	The Lancet. Oncology 2021;22:7:1023-1033
Study of mirtazapine for agitated behaviours in dementia (SYMBAD): a randomised, double-blind, placebo-controlled trial.	Lancet 2021;398:10310:1487-1497
Subcutaneous REGEN-COV Antibody Combination to Prevent Covid-19.	The New England journal of medicine 2021;385:13:1184-1195
Subcutaneous Rituximab-MiniCHOP Compared With Subcutaneous Rituximab-MiniCHOP Plus Lenalidomide in Diffuse Large B-Cell Lymphoma for Patients Age 80 Years or Older.	Journal of clinical oncology 2021;39:11:1203-1213
Supervised versus unsupervised primaquine radical cure for the treatment of falciparum and vivax malaria in Papua, Indonesia: a cluster-randomised, controlled, open-label superiority trial.	The Lancet. Infectious diseases 2022;22:3:367-376
Surgery for malignant pleural mesothelioma after radiotherapy (SMART): final results from a single-centre, phase 2 trial.	The Lancet. Oncology 2021;22:2:190-197
Systematic monitoring for detection of atrial fibrillation in patients with acute ischaemic stroke (MonDAFIS): a randomised, open-label, multicentre study.	The Lancet. Neurology 2021;20:6:426-436
Targeting Depressive Symptoms in Younger Breast Cancer Survivors: The Pathways to Wellness Randomized Controlled Trial of Mindfulness Meditation and Survivorship Education.	Journal of clinical oncology 2021;39:31:3473-3484
Ten-Year Outcome of Neoadjuvant Chemoradiotherapy Plus Surgery for Esophageal Cancer: The Randomized Controlled CROSS Trial.	Journal of clinical oncology 2021;39:18:1995-2004
Terlipressin plus Albumin for the Treatment of Type 1 Hepatorenal Syndrome.	The New England journal of medicine 2021;384:9:818-828
Tezepelumab in Adults and Adolescents with Severe, Uncontrolled Asthma.	The New England journal of

	medicine 2021;384:19:1800-1809
The Effect of Flat Flexible Versus Stable Supportive Shoes on Knee Osteoarthritis Symptoms : A Randomized Trial.	Annals of internal medicine 2021;174:4:462-471
The Effects of Four Doses of Vitamin D Supplements on Falls in Older Adults : A Response-Adaptive, Randomized Clinical Trial.	Annals of internal medicine 2021;174:2:145-156
The Randomized AMBORA Trial: Impact of Pharmacological/Pharmaceutical Care on Medication Safety and Patient-Reported Outcomes During Treatment With New Oral Anticancer Agents.	Journal of clinical oncology 2021;39:18:1983-1994
The Safety and Immunologic Effectiveness of the Live Varicella-Zoster Vaccine in Patients Receiving Tumor Necrosis Factor Inhibitor Therapy : A Randomized Controlled Trial.	Annals of internal medicine 2021;174:11:1510-1518
The SANAD II study of the effectiveness and cost-effectiveness of valproate versus levetiracetam for newly diagnosed generalised and unclassifiable epilepsy: an open-label, non-inferiority, multicentre, phase 4, randomised controlled trial.	Lancet 2021;397:10282:1375-1386
The Trial to Reduce Antimicrobial Use in Nursing Home Residents With Alzheimer Disease and Other Dementias (TRAIN-AD): A Cluster Randomized Clinical Trial.	JAMA internal medicine 2021;181:9:1174-1182
The Use of Rifaximin in the Prevention of Overt Hepatic Encephalopathy After Transjugular Intrahepatic Portosystemic Shunt : A Randomized Controlled Trial.	Annals of internal medicine 2021;174:5:633-640
Therapeutic Anticoagulation with Heparin in Critically Ill Patients with Covid-19.	The New England journal of medicine 2021;385:9:777-789
Therapeutic Anticoagulation with Heparin in Noncritically Ill Patients with Covid-19.	The New England journal of medicine 2021;385:9:790-802

Ticagrelor versus Clopidogrel in CYP2C19 Loss-of-Function Carriers with Stroke or TIA.	The New England journal of medicine 2021;385:27:2520-2530
Tocilizumab in Patients Hospitalized with Covid-19 Pneumonia.	The New England journal of medicine 2021;384:1:20-30
Tofacitinib in juvenile idiopathic arthritis: a double-blind, placebo-controlled, withdrawal phase 3 randomised trial.	Lancet 2021;398:10315:1984-1996
Tofacitinib in Patients Hospitalized with Covid-19 Pneumonia.	The New England journal of medicine 2021;385:5:406-415
Total Body Irradiation or Chemotherapy Conditioning in Childhood ALL: A Multinational, Randomized, Noninferiority Phase III Study.	Journal of clinical oncology 2021;39:4:295-307
Toxicity Index, Patient-Reported Outcomes, and Early Discontinuation of Endocrine Therapy for Breast Cancer Risk Reduction in NRG Oncology/NSABP B-35.	Journal of clinical oncology 2021;39:34:3800-3812
Tranexamic Acid for the Prevention of Blood Loss after Cesarean Delivery.	The New England journal of medicine 2021;384:17:1623-1634
Transdermal oestradiol for androgen suppression in prostate cancer: long-term cardiovascular outcomes from the randomised Prostate Adenocarcinoma Transcutaneous Hormone (PATCH) trial programme.	Lancet 2021;397:10274:581-591
TRANSFORMER: A Randomized Phase II Study Comparing Bipolar Androgen Therapy Versus Enzalutamide in Asymptomatic Men With Castration-Resistant Metastatic Prostate Cancer.	Journal of clinical oncology 2021;39:12:1371-1382
Trastuzumab deruxtecan (DS-8201) in patients with HER2-expressing metastatic colorectal cancer	The Lancet. Oncology

(DESTINY-CRC01): a multicentre, open-label, phase 2 trial.	2021;22:6:779-789
Trastuzumab Emtansine Plus Pertuzumab Versus Taxane Plus Trastuzumab Plus Pertuzumab After Anthracycline for High-Risk Human Epidermal Growth Factor Receptor 2-Positive Early Breast Cancer: The Phase III KAITLIN Study.	Journal of clinical oncology 2022;40:5:438-448
Trial of Intensive Blood-Pressure Control in Older Patients with Hypertension.	The New England journal of medicine 2021;385:14:1268-1279
Trial of Pimavanserin in Dementia-Related Psychosis.	The New England journal of medicine 2021;385:4:309-319
Trial of Psilocybin versus Escitalopram for Depression.	The New England journal of medicine 2021;384:15:1402-1411
Trial of Spesolimab for Generalized Pustular Psoriasis.	The New England journal of medicine 2021;385:26:2431-2440
Triple Therapy for Cystic Fibrosis Phe508del-Gating and -Residual Function Genotypes.	The New England journal of medicine 2021;385:9:815-825
Ultra-early tranexamic acid after subarachnoid haemorrhage (ULTRA): a randomised controlled trial.	Lancet 2021;397:10269:112-118
Ultrasound renal denervation for hypertension resistant to a triple medication pill (RADIANCE-HTN TRIO): a randomised, multicentre, single-blind, sham-controlled trial.	Lancet 2021;397:10293:2476-2486

Updated Overall Survival and PD-L1 Subgroup Analysis of Patients With Extensive-Stage Small-Cell Lung Cancer Treated With Atezolizumab, Carboplatin, and Etoposide (IMpower133).	Journal of clinical oncology 2021;39:6:619-630
Use of cast immobilisation versus removable brace in adults with an ankle fracture: multicentre randomised controlled trial.	BMJ 2021;374:n1506
Use of High-Sensitivity Cardiac Troponin in Patients With Kidney Impairment: A Randomized Clinical Trial.	JAMA internal medicine 2021;181:9:1237-1239
Use of metformin to prolong gestation in preterm pre-eclampsia: randomised, double blind, placebo controlled trial.	BMJ 2021;374:n2103
Use of proton pump inhibitors to treat persistent throat symptoms: multicentre, double blind, randomised, placebo controlled trial.	BMJ 2021;372:m4903
Vaccine efficacy against persistent human papillomavirus (HPV) 16/18 infection at 10 years after one, two, and three doses of quadrivalent HPV vaccine in girls in India: a multicentre, prospective, cohort study.	The Lancet. Oncology 2021;22:11:1518-1529
Vaccine Efficacy of ALVAC-HIV and Bivalent Subtype C gp120-MF59 in Adults.	The New England journal of medicine 2021;384:12:1089-1100
Vagus nerve stimulation paired with rehabilitation for upper limb motor function after ischaemic stroke (VNS-REHAB): a randomised, blinded, pivotal, device trial.	Lancet 2021;397:10284:1545-1553
Veliparib in Combination With Platinum-Based Chemotherapy for First-Line Treatment of Advanced Squamous Cell Lung Cancer: A Randomized, Multicenter Phase III Study.	Journal of clinical oncology 2021;39:32:3633-3644
Web-Based Educational Intervention for Patients With Uninvestigated Dyspepsia Referred for Upper Gastrointestinal Tract Endoscopy: A Randomized Clinical Trial.	JAMA internal medicine 2021;181:6:825-833
